# The role of intestinal homeostasis in sevoflurane-induced myelin development and cognitive impairment in neonatal mice

**DOI:** 10.3389/fcimb.2025.1541757

**Published:** 2025-03-12

**Authors:** Chang Liu, Jinjie Li, Ruizhu Liu, Guoqing Zhao

**Affiliations:** ^1^ Department of Anesthesiology, China-Japan Union Hospital of Jilin University, Changchun, China; ^2^ Jilin University, Changchun, China

**Keywords:** sevoflurane, neonate, gut microbiota, short-chain fatty acid, myelin

## Abstract

**Background:**

Inhalational anesthetic sevoflurane is commonly used in pediatric anesthesia. Multiple exposures to sevoflurane in early postnatal life have been associated with long-term abnormalities in myelin development and cognitive and memory impairments, although the underlying mechanisms remain incompletely elucidated. Disruption of gut microbiota is recognized as an important contributor to neurological diseases. Here, we explore the potential mechanisms underlying the abnormal myelin development induced by multiple sevoflurane exposures in neonatal rats by analyzing gut homeostasis.

**Methods:**

Six-day-old (P6) C57BL/6 mice were exposed to 3% sevoflurane for 2 hours per day for three consecutive days. Mice exposed to a mixture of 60% nitrogen and oxygen under the same conditions and duration served as controls. Behavioral tests were conducted between P32 and P42. At P9 (24 hours after the last sevoflurane exposure) and P42 (after the completion of behavioral tests), intestinal and brain examinations were performed to investigate the effects of sevoflurane exposure during the lactation and adolescent periods on gut homeostasis and myelin development in mice. Subsequently, the ameliorative effects of butyrate supplementation on sevoflurane-induced abnormalities in myelin development and cognitive and memory impairments were observed.

**Results:**

After repeated exposure to sevoflurane, neonatal mice developed persistent gut microbiota imbalance accompanied by a decrease in short-chain fatty acids. Short-term intestinal inflammation emerged, with damage to the mucus layer and barrier function. In the hippocampus and prefrontal cortex, the expression of genes and transcription factors related to oligodendrocyte differentiation and myelin development was significantly affected, and these changes persisted even after the exposure ended. There was a reduction in proteins associated with oligodendrocytes and myelin formation, which had a certain impact on memory and cognitive behavior. This study also explored the potential connections between microbiota, metabolism, the gut, the brain, and behavior. Timely supplementation with butyrate could effectively reverse these changes, indicating that gut homeostasis is crucial for brain neurodevelopment.

**Conclusion:**

Multiple exposures to sevoflurane in neonatal mice disrupt gut homeostasis and affect oligodendrocyte differentiation and myelin development in the hippocampus and prefrontal cortex, inducing cognitive and memory impairments. Supplementation with butyrate can alleviate these changes.

## 1 Introduction

General anesthesia, as a crucial component of life support in modern medicine, is administered to millions of infants and young children worldwide each year due to treatment needs (Weiser et al., 2008). Inhalation anesthesia is widely used in clinical practice for infants and young children due to its advantages of rapid onset, controllable depth of anesthesia, high safety, and reduction of psychological trauma (Wang et al., 2020).

However, a growing body of clinical and experimental evidence suggests that inhalational anesthetics, such as sevoflurane, may have lasting effects on the developing brain. Epidemiological studies have shown that children who undergo general anesthesia before the age of 3 are at a significantly increased risk of developing language and cognitive impairments by the age of 10-12 (Ing et al., 2012). Clinical cohort studies further confirm that multiple exposures to sevoflurane are associated with delayed neurocognitive development in later childhood (Wang et al., 2014). Animal experiments also indicate that neonatal exposure to sevoflurane can lead to impaired synaptic plasticity in the hippocampus, accompanied by long-term deficits in spatial learning and memory function (Zhao et al., 2022). These findings have raised profound concerns about the risk of persistent cognitive impairment that young children may face following inhalational anesthesia.

Myelin serves as the primary regulator of axonal conduction in the central nervous system, and its integrity is crucial for the normal development and plasticity of central nervous system functions (Monje, 2018). Notably, the critical window for neural myelin development in the brain occurs early in life (Grotheer et al., 2022). Clinical studies have shown that the greater the drug dosage and the longer the cumulative duration of sevoflurane anesthesia exposure in pediatric patients, the more significant the potential impact on the integrity of the corpus callosum white matter (Banerjee et al., 2019). Additionally, experiments in rodent models have revealed potential toxic effects of sevoflurane on myelin development (Wu et al., 2020). However, the underlying mechanisms remain incompletely understood.

In recent years, research into the mechanisms underlying the interaction between the gut microbiota and the central nervous system has provided a new perspective for understanding the neurotoxicity of anesthetic drugs. An increasing number of studies have shown that exposure to sevoflurane significantly affects the composition and function of the gut microbiota. For instance, Liu et al. found that sevoflurane exposure alters the composition of the gut microbiota in neonatal rats (Liu et al., 2021). Han et al. administered 4 hours of sevoflurane inhalational anesthesia to mice and collected feces from both experimental and control groups on days 1, 3, 7, and 14 post-anesthesia. They discovered that sevoflurane inhalational anesthesia induces changes in the gut microbiome of mice, which begin to appear on day 1 post-anesthesia and reach their most significant level on day 7 (Han et al., 2021). Additionally, studies have shown that early repeated exposure to sevoflurane anesthesia and surgery can lead to gut microbiota dysregulation after 4 weeks, manifested by a decrease in the relative abundance of Lactobacillaceae and an increase in the relative abundance of Verrucomicrobiaceae (Zhou et al., 2023). Adult mice also exhibit abnormal gut microbiota composition following anesthesia and surgery, accompanied by delirium-like behavior. There are significant differences in 20 gut bacteria between mice with and without delirium. Germ-free mice that received the gut microbiota from post-operative delirium mice exhibited similar behavior, suggesting that changes in the gut microbiota are associated with delirium behavior (Zhang et al., 2019). Another preclinical study revealed that neonatal rats exposed to a single inhalational anesthetic during the neonatal period undergo significant changes in their gut microbiota, with an increase in the abundance of Firmicutes and Lachnospiraceae and a decrease in the abundance of Bacteroidetes and Bacteroidaceae compared to controls (Wang et al., 2019). Furthermore, researchers using germ-free mice have found that the microbiota is necessary for myelination and maintaining myelin plasticity (Keogh et al., 2021).

Bacterial metabolites, short-chain fatty acids (SCFAs), have demonstrated significant effects in regulating organismal health. Studies have shown that SCFAs are effective in maintaining gut health (Makki et al., 2018; Morrison and Preston, 2016). They not only alleviate stress-induced behavioral deficits and repair functional disorders of the intestinal barrier (van de Wouw et al., 2018) but also possess the ability to regulate myelination in the prefrontal cortex of the brain (Gacias et al., 2016). Moreover, they have been proven to beneficially regulate brain function and behavioral performance (Gacias et al., 2016). These groundbreaking discoveries have revealed a novel and crucial interaction pattern of the microbe-gut-brain axis (MGBA).

Given the widespread use of sevoflurane in pediatric anesthesia and its potential long-term neurodevelopmental effects, conducting in-depth research on its mechanisms of impact on neurodevelopment and exploring effective intervention strategies hold significant social value for ensuring the healthy growth of children and optimizing anesthesia protocols. Therefore, this study aims to investigate the short- and long-term effects of sevoflurane exposure on gut homeostasis and neuromyelin development in neonatal mice, and to explore the underlying mechanisms of the MGBA. The ultimate goal is to provide new theoretical foundations and intervention strategies for the prevention and treatment of anesthesia-related neurodevelopmental disorders.

## 2 Materials and methods

### 2.1 Animals

Thirty-three C57BL/6J mice on the 18th day of gestation were purchased from the Animal Center of the School of Public Health, Jilin University, and housed in a specific pathogen-free animal facility. The environmental temperature in the animal room was maintained at 22-24°C, with a humidity level of 40%-50%. The animal room followed a 12-hour light-dark cycle (lights on from 7:00 to 19:00) to ensure normal circadian rhythms in the mice. Food and drinking water were provided in sufficient quantities, allowing the mice to feed freely. On the fifth day after the birth, male pups from the litter were selected as experimental subjects and were randomly assigned to different groups. All animal procedures in this study strictly adhered to the “Guide for the Care and Use of Laboratory Animals,” and all research protocols involving mice were reviewed and approved by the Animal Care and Use Committee of Jilin University (Changchun, Jilin, China) (Ethical Protocol Number: SY: 2024-06-012). No surgeries were performed, and all efforts were made to minimize animal suffering and reduce the number of animals used.

### 2.2 Study design

In this study, a total of 100 six-day-old male C57BL/6 pups were used and randomly assigned to the following eight groups: Control_Lactation Period (Con_LP, n=20), Sevoflurane_Lactation Period (Sev_LP, n=20), Control_Adolescence (Con_Adol, n=10), Sevoflurane_Adolescence (Sev_Adol, n=10), Control_Normal Saline (Con_NS, n=10), Control_Sodium Butyrate (Con_NaB, n=10), Sevoflurane_Normal Saline (Sev_NS, n=10), and Sevoflurane_Sodium Butyrate (Sev_NaB, n=10).

The Sev_LP, Sev_Adol, Sev_NS, and Sev_NaB groups were exposed to 3 vol% sevoflurane for 2 hours each day from P6 to P8, for three consecutive days, conceptually mimicking multiple exposures to anesthesia in infants and young children (Zhang et al., 2023). The Con_LP, Con_Adol, Con_NS, and Con_NaB groups were exposed to a mixture of 60% nitrogen and oxygen for 2 hours each day during the same period. The Con_LP and Sev_LP groups were euthanized on P9. Behavioral experiments were conducted on the Con_Adol and Sev_Adol groups from P32 to P42, followed by euthanasia. Con_NS and Sev_NS received saline gavage once a day for three weeks after the end of sevoflurane exposure, with behavioral experiments conducted from P34 to P42, followed by euthanasia. Con_NaB and Sev_NaB received 300mg/kg sodium butyrate gavage therapy once a day for three weeks after the end of sevoflurane exposure, with behavioral experiments conducted from P34 to P42, followed by euthanasia. The behavioral testing for Con_NS, Sev_NS, Con_NaB, and Sev_NaB groups starting from P34 was based on the fact that no anxiety-like behavior differences were detected between Con_Adol and Sev_Adol groups in the open field test (OFT) on P32 and the elevated plus maze test (EPM) on P33. Therefore, these two tests were not conducted again subsequently, and memory and cognitive function assessment experiments were directly initiated from P34.

### 2.3 Sevoflurane exposure

Neonatal mice were exposed to 3% sevoflurane for 2 hours each day from P6 to P8, consecutively for three days. The exposure to the volatile anesthetic was conducted using a RWD R630 enhanced small animal anesthesia machine. The mice were induced in a plexiglass chamber connected to the vaporizer, with a mixture of 3 vol% sevoflurane and 60% nitrogen-oxygen gas at an oxygen flow rate of 5L/min for 3-5 minutes, until the righting reflex disappeared. Subsequently, the oxygen flow rate was reduced to 2L/min to maintain anesthesia for 2 hours. During the sevoflurane anesthesia, concentrations of sevoflurane, oxygen, and carbon dioxide were monitored using an infrared probe (OhmedaS/5 Compact, Datex-Ohmeda, Louisville, CO, USA). Heart rate and body temperature were monitored using a monitor (Jeruitai, GT6800VET, Changsha, China). A heating pad was used to maintain a constant body temperature of the mice at 36.5 ± 0.5°C. After the sevoflurane exposure, the mice were returned to their home cages with their littermates once the righting reflex was restored. All animals (100%) survived the sevoflurane exposure.

### 2.4 Behavior tests

#### 2.4.1 Open field test (OFT)

The study employed the OFT to assess the spontaneous behavior, exploratory behavior, and anxiety levels of the mice in a novel environment (P32). The mice were placed in the testing room 30 minutes prior to the test to acclimate to the room temperature and humidity. Before the formal experiment, each mouse was placed alone in a large empty box for 5 minutes to adapt to the open environment. The room was kept quiet during the experiment. The mouse was then removed from the box and placed in the central area of the open field apparatus, facing away from the experimenter. The experimenter quickly left the room, and the system automatically recorded the mouse’s activity and related parameters for 15 minutes. After 15 minutes, the mouse was removed, and the entire open field apparatus was cleaned with 75% alcohol to eliminate any residual information (such as feces, urine, or odor) from the previous mouse, which could affect the next test result. The process was repeated for each mouse. In this study, a ceiling-mounted video system (Jiangsu Sai’angsi Biotechnology Co., Ltd.) was used to record the movement trajectories, and the SANS animal behavior video analysis software was utilized to calculate the distance traveled in the central area, the total distance traveled, and the time spent in the central area.

#### 2.4.2 Elevated Plus-Maze Test (EPM)

The study employed the EPM to assess anxiety-related behavior in mice (P33). At the start of the experiment, each mouse was placed on the central platform facing one of the open arms and introduced into the maze. The activity of the mouse was recorded for 5 minutes. After each mouse was tested, the maze was wiped with 75% alcohol to avoid any scent traces. The SANS animal behavior video analysis software was used to record and measure the time spent in the open arms and the total number of entries into the open arms to assess behaviors related to anxiety.

#### 2.4.3 Object Location Test (OLT) and Novel Object Recognition Test (NORT)

**Figure 1:**
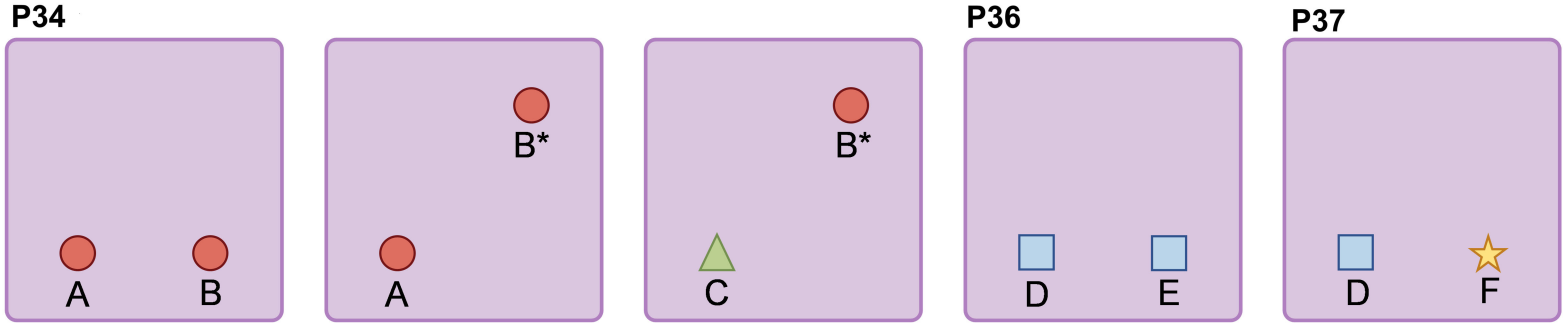
Schematic diagram of OLT and NORT.

The study employed OLT and NORT to assess the short-term and long-term memory of the mice (P34-P37). The mice were placed in the testing room 30 minutes prior to the experiments. During the familiarization phase, the mice were placed in the open field arena and allowed to freely explore for 6 minutes. This was followed by a 20 minutes rest period, and the process was repeated three times. For the testing phase, as illustrated in **Figure 1**, objects A and B were placed 7 cm away from the edges of the open field arena. The mice were then introduced into the arena to explore both objects for 10 minutes. After being removed and given a 30 minutes rest, object B was moved to a new position B*, and the mice were again introduced into the arena for another 10 minutes exploration. Following another 30 minutes rest, object A was replaced with a novel object C, and the mice were introduced into the arena for a final 10 minutes exploration. After a one-day break, the mice were introduced into the arena to explore two new objects D and E for 10 minutes. On the following day, object E was replaced with another novel object F, and the mice were allowed to explore objects D and F for 10 minutes. After each session, the open field arena was wiped with 75% alcohol during the mice’s rest intervals to eliminate any residual odors or cues. The exploration time of each mouse for each object was recorded using a video camera and analyzed using the SANS animal behavior video analysis software.

#### 2.4.4 Morris water maze(MWM)test

The study employed the MWM test to assess the learning and memory abilities of mice (P38-P42). In the MWM test, the escape platform was positioned 1 centimeter below the water surface. During the 4-day place navigation experiment, if a mouse successfully found the platform within 60 seconds, it was allowed to remain on the platform for 10 seconds. If the mouse failed to find the platform within 90 seconds, it was guided to the platform by the experimenter and remained there for 30 seconds. On the 5th day, a spatial probe test was conducted, where the escape platform was removed and the trained mice were allowed to explore the maze for 60 seconds. After the test, each mouse was placed in a warming cage to dry for 5 minutes before being returned to its home cage. The swimming trajectories were tracked and recorded using a ceiling-mounted camera and a computer system equipped with behavioral video analysis software (SANS, Jiangsu Sai’angsi Biotechnology Co., Ltd.). The escape latency, platform crossing frequency, and time spent in the target quadrant were calculated as indicators of learning and memory function.

### 2.5 Sample collection

After weighing the P9 pups, they were anesthetized with 3% sevoflurane for 3-5 minutes and euthanized by rapid decapitation. The colonic contents were collected into sterile cryotubes. Due to the small amount of intestinal content, the contents from three pups were combined into one sample. Additionally, colonic tissue, prefrontal cortex, and hippocampal tissue were collected, frozen in liquid nitrogen for 30 minutes, and then stored at -80°C. For the P42 mice, after completing the behavioral experiments, they were weighed, anesthetized with 3% sevoflurane for 3-5 minutes, and euthanized by rapid decapitation. The colonic contents, colonic tissue, prefrontal cortex, and hippocampal tissue were collected into sterile cryotubes, frozen in liquid nitrogen for 30 minutes, and then stored at -80°C.

### 2.6 16S rRNA sequencing analysis

Total DNA was extracted from the fecal microbiota of mice using the BIOG DNA Stool Kit (Changzhou, China) according to the manufacturer’s instructions. DNA quantification was performed using a Nanodrop, and the quality of DNA extraction was assessed by 1.2% agarose gel electrophoresis. PCR amplification was performed targeting the V3-V4 region of the 16S rRNA gene of microorganisms using primers 338F (5’-ACTCCTACGGGAGGCAGCA-3’) and 806R (5’-GGACTACHVGGGTWTCTAAT-3’).The amplified products were purified and recovered using magnetic beads (Vazyme VAHTSTM DNA Clean Beads) at 0.8 times the volume of the 25 μl PCR product. Subsequently, the recovered PCR amplification products were quantified using the Quant-iT PicoGreen dsDNA Assay Kit with a Microplate reader (BioTek, FLx800). Based on the fluorescence quantification results, the samples were mixed in appropriate proportions. The sequencing library was prepared using Illumina’s TruSeq Nano DNA LT Library Prep Kit, with a sequencing depth of 50,000 reads per sample. After sequencing, OTU clustering was performed following the QIIME2 dada2 analysis workflow. The Alpha diversity level of each sample was assessed based on the distribution of ASVs/OTUs across different samples, and rarefaction curves were used to evaluate the adequacy of sequencing depth. At the ASV/OTU level, distance matrices were calculated for each sample, and unsupervised ordination, clustering, and other methods were employed to measure beta diversity differences between samples. At the species taxonomic composition level, unsupervised and supervised ordination, clustering, and modeling approaches were utilized to further assess differences in species abundance composition between samples and attempt to identify indicator species. Screening for biomarker species was conducted using Lefse analysis (LDA effect size > 3, P-value < 0.05). Based on the species composition distribution across samples, association networks were constructed, topological indices were calculated, and efforts were made to identify key species.

### 2.7 Gas chromatography–mass spectrometry (GC–MS) analysis of SCFAs

After homogenization, the mouse fecal samples were resuspended in pure water at a 1:1 ratio and then centrifuged at 12,000 rpm for 15 minutes at 4°C to remove insolubles. Next, 100 μL of 15% phosphoric acid solution and 20 μL of isohexanoic acid solution (375 μg/mL) (as an internal standard) were added to the centrifuged supernatant (200 μL), followed by the addition of 280 μL of ether for extraction. The mixture was then centrifuged again at 12,000 rpm for 10 minutes at 4°C to separate the organic phase. Subsequently, the supernatant was analyzed by GC-MS (Thermo, USA), with QC samples used for quality control. After detecting a series of standard solution concentrations, a calibration curve was constructed with the concentration of the standard as the abscissa and the peak area ratio of the standard to the internal standard as the ordinate. The standard curve was used to quantify short-chain fatty acids. Data scaling and clustering analysis were performed on the dataset using the Pheatmap package in R (v4.0.3).

### 2.8 Histological analysis

#### 2.8.1 Histological staining

Mouse brain and intestinal tissue samples were fixed in 4% paraformaldehyde (PFA). The tissue samples were dehydrated through a series of graded ethanols, embedded in paraffin, and sectioned into 5-micrometer thick slices using a microtome (Leica, Germany). Subsequently, the colonic sections were stained with Hematoxylin-Eosin(HE) and Alcian Blue(AB), while the prefrontal cortex and hippocampus regions of the brain were stained with HE and Luxol Fast Blue(LFB). These images were observed and captured using an optical microscope (Nikon, Japan), and quantitative analysis was performed using ImageJ software (v 1.47, Bethesda, MD).

#### 2.8.2 Transmission Electron Microscope (TEM)

The mouse colon tissue specimens were trimmed into 1mm x 1mm x 3mm blocks and quickly placed in electron microscopy fixative (a mixture of 3% paraformaldehyde and 1% glutaraldehyde (4°C, pH 7.4)) for 2 hours at room temperature in the dark, then transferred to 4°C for storage. The tissue blocks were then placed in 2% glutaraldehyde phosphate-buffered fixative and stored overnight at 4°C. After rinsing with PBS buffer, the samples were post-fixed with osmic acid for 1.5 hours, dehydrated through a series of graded ethanols, and infiltrated with epoxy propane for 15 minutes. The samples were then embedded in embedding medium and polymerized at 60°C for 24 hours. Observation and imaging were performed using a Hitachi-7500 TEM with an accelerating voltage of 80kV.

#### 2.8.3 Immunofluorescence (IF)

Mouse brain and intestinal tissue samples were fixed in 4% PFA. The tissue samples were dehydrated through a series of graded ethanols, embedded in paraffin, and sectioned into 20-micrometer thick slices using a microtome (Leica, Germany). The tissue sections were placed in a repair box containing EDTA antigen retrieval buffer (pH 8.0) for antigen retrieval. After briefly drying the sections, a histochemical pen was used to draw a circle around the tissue, and then spontaneous fluorescence quencher was added and incubated for 5 minutes, followed by rinsing with running water for 10 minutes. After blocking with 5% BSA normal goat serum (for 1 hour at room temperature), the samples were incubated with primary antibodies overnight at 4°C (for 16 hours). Following washing with PBS buffer, the samples were incubated with appropriate secondary antibodies at room temperature for 1 hour, then washed with PBS buffer, and stained with DAPI (Southern Biotech, AL, USA) at 22°C for 2 minutes. The slides were mounted with Prolong diamond (Invitrogen), and images were captured using a confocal microscope (A1R-SIMSTORM, Nikon, Japan). Quantitative analysis was performed using Image J software (v 1.47, Bethesda, MD). The antibodies used are listed in **Supplementary Table 1**.

### 2.9 Quantitative real-time PCR analysis

Total RNA was isolated from mouse intestinal and brain tissue samples using Trizol reagent (Ambion, USA) according to the manufacturer’s protocol. The purity of the total RNA was measured using a NanoDrop spectrophotometer (Thermo Fisher Scientific, Beverly, MA). Then, cDNA samples were synthesized using the HiScript III RT SuperMix for qPCR (+gDNA wiper) (Vazyme) according to the manufacturer’s instructions. Quantitative analysis was performed using SYBR green dye on a QuantStudio 6 Flex Real-Time PCR System (Applied Biosystems) with the SYBR Premix Ex Taq II kit (Takara, Dalian, China) on a Bio-Rad CFX384. Specific primer pairs were synthesized by Sangon Biotech (Shanghai, China) and are listed in **Supplementary Table 2**. β-Actin was used as an internal reference gene, and data were analyzed using the 2-ΔΔCT method to calculate the relative gene expression fold change for each tissue sample.

### 2.10 Statistical analysis

The Shapiro-Wilk test was used to check for normality. Continuous variables that followed a normal distribution were expressed as the mean ± standard deviation (SD). Statistical analyses were performed using IBM SPSS Statistics v.24.0 and GraphPad Prism v.7 software. Differences in normally or non-normally distributed data were measured using Student’s t-test or the Mann-Whitney U test, respectively. Comparisons between multiple groups were conducted using one-way or two-way analysis of variance (ANOVA). Differences were considered statistically significant when p < 0.05. Spearman’s rank correlation analysis was used to determine correlations.

## 3 Results

### 3.1 Long-term changes in gut microbiota composition induced by repeated sevoflurane exposure in infant rats

**Figure 2:**
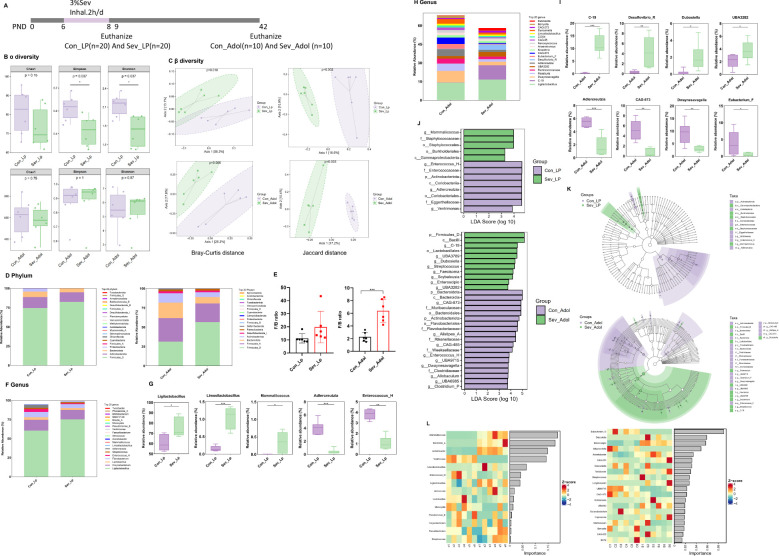
Effects of Sevoflurane Exposure on Gut Microbiota in Neonatal Rats During the Suckling and Adolescent Periods. **(A)** Timeline of sevoflurane exposure and gut microbiota analysis. **(B)** Analysis of Chao1 index, Simpson index, and Shannon index in Alpha diversity. **(C)** Beta diversity analysis using Bray-Curtis distance and Jaccard distance. **(D)** Abundance of gut microbiota composition at the phylum level. **(E)** Ratio of Firmicutes to Bacteroidetes (F/B). **(F)** Abundance of gut microbiota composition at the genus level in suckling rats. **(G)** Statistically significant bacterial communities at the genus level in suckling rats. **(H)** Abundance of gut microbiota composition at the genus level in adolescent rats. **(I)** Statistically significant bacterial communities at the genus level in adolescent rats. **(J)** LDA score bar chart of biomarker species in LEfSe analysis. The vertical axis represents bacteria with significant differences between groups, and the horizontal axis shows the logarithm of the LDA score for each taxonomic unit. **(K)** Phylogenetic tree of differential species annotations in LEfSe. **(L)** Random forest plot. The statistic difference of various indicators between two groups was evaluated by two-tailed unpaired student’s T test and When p < 0.05, it is considered statistically significant. The significance levels are represented as: *P<0.05, **P<0.01, ***P<0.001, ****P<0.0001, while ns stands for no significant difference.

At two time points, P9 and P42, we analyzed the characteristics of gut microbiota composition in mice through 16S rRNA sequencing to observe the short-term and long-term effects of sevoflurane exposure (**Figure 2A**).

Alpha diversity reflects the abundance, diversity, and evenness of the microbial community. As shown in **Figure 2B**, there were no significant differences in the Chao1 index between groups during both the suckling and adolescent periods, indicating no significant change in the total number of species. In the suckling period, the Simpson index and Shannon index were relatively lower in the sevoflurane-exposed group compared to the control, whereas there were no significant differences during adolescence. This suggests that sevoflurane exposure temporarily reduces the diversity of the gut bacterial community in mice, but it recovers during adolescence.

Further beta diversity analysis was conducted to assess the differences and similarities in gut microbiota structure between different groups. Based on unsupervised PCoA analysis using Bray-Curtis distance and Jaccard distance, as shown in **Figure 2C**, significant differences were observed in both Bray-Curtis distance and Jaccard distance between groups during both the suckling and adolescent periods. This indicates that sevoflurane exposure induces significant changes in the species structure and composition of the gut microbiota in mice, and these changes persist into adolescence.

**Figure 2D** illustrates the relative abundance composition of gut microbiota at the phylum level. During the suckling period, the species composition is dominated by Firmicutes_D and Actinobacteria, together accounting for approximately 90% of the total bacterial relative abundance. At this stage, the abundance of various bacteria in the mouse gut is low, and the gut microbiome is not yet mature. In adolescence, the species composition is primarily composed of Firmicutes_D, Firmicutes_A, Bacteroidetes, and Actinobacteria, collectively accounting for over 90% of the total bacterial relative abundance. At this stage, the richness of various bacteria in the mouse gut increases, and the gut microbiome is basically mature. Compared to the control group, the abundance of Firmicutes in the sevoflurane-exposed group increased from 67.7% to 80.7%, with Firmicutes_D being the main contributor to this increase, while Firmicutes_A showed a decrease. The abundance of Bacteroidetes decreased from 14% to 8%, and Actinobacteria also decreased from 12% to 6%. The Firmicutes/Bacteroidetes (F/B) ratio was significantly higher in the experimental group compared to the control group (t=5.157,p=0.0004) (**Figure 2E**), indicating a higher abundance of Gram-positive bacteria in the experimental group.

At the genus level, more detailed changes in the bacterial community can be observed (**Figures 2F, H**). During the suckling period, compared to the control group, the sevoflurane-exposed group showed an increase in the genera Ligilactobacillus, Limosilactobacillus, and Mammaliicoccus (t=3.012,p=0.013) (t=6.928,p<0.0001)(t=2.723,p=0.022), while the genera Enterococcus_H and Adlercreutzia decreased (t=7.893,p<0.0001)(t=3.206,p=0.0013) (**Figure 2G**). In adolescence, the changes at the genus level were even more pronounced (**Figure 2H**). Compared to the control group, the sevoflurane-exposed group had an increase in the relative abundance of the genera C-19, UBA3282, Desulfovibrio_R, and Dubosiella (t=6.818,p<0.0001)(t=2.040,p=0.049)(t=3.294,p=0.0081)(t=2.765,p=0.02), while the genera Dwaynesavagella, Adlercreutzia, Eubacterium_F, and CAG-873 decreased (t=3.450,p=0.0062)(t=5.496,p=0.0003)(t=2.108,p=0.049)(t=5.149,p=0.004)(**Figure 2I**). In summary, repeated sevoflurane exposure in neonates leads to long-term changes in the composition of the gut bacterial community.

Through LEfSe analysis, we screened for the most significantly changed taxonomic units between groups (LDA effect size > 3, P-value < 0.05)(**Figure 2J**). We found that during the suckling period, the sevoflurane-exposed group had an increase in bacteria subclassified under p:Firmicutes_D, including g:Mammaliicoccus, f:Staphylococcaceae, o:Staphylococcales, as well as an increase in bacteria subclassified under Proteobacteria, including o:Burkholderiales, c:Gammaproteobacteria. In the control group, there was an increase in bacteria subclassified under Actinobacteria, including c:Coriobacteriia, o:Coriobacteriales, f:Eggerthellaceae, g:Adlercreutzia. In adolescence, the sevoflurane-exposed group had an increase in p:Firmicutes_D and its subclassified bacteria, including c:Bacilli, g:C-19, o:Lactobacillales, etc. The control group had an increase in bacteria subclassified under Bacteroidetes, including c:Bacteroidia, g:CAG-873, f:Muribaculaceae, etc., as well as an increase in p:Actinobacteriota. **Figure 2K** is a cladogram of differential species annotations generated by the LEfSe method, where nodes representing colors corresponding to different groups indicate significantly different species with relatively higher abundance in that group.

Random forest analysis revealed that during the suckling period, Mammaliicoccus, Bordetella_A, Acinetobacter, Ventrimonas, and Limosilactobacillus are the top five most important biomarkers for distinguishing between the two groups. In adolescence, Eubacterium_G, Dubosiella, Enteroscipio, Alloprevotella, and Acinetobacter are the top five most important biomarkers for distinguishing between the two groups (**Figure 2L**).

Overall, our results indicate that repeated exposure to inhalational anesthetics during the neonatal period disrupts the gut microbiota, and this alteration persists into adolescence.

### 3.2 Long-term changes in SCFAs induced by repeated sevoflurane exposure in neonatal mice

**Figure 3:**
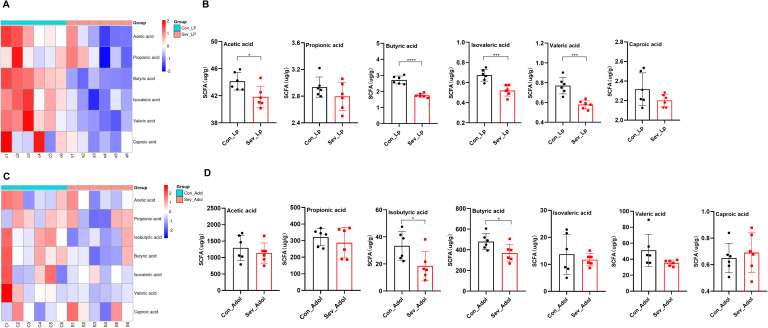
Effects of sevoflurane exposure on gut SCFAs in neonatal mice during lactation and adolescence. **(A)** Clustering heatmap of gut SCFAs in mice during lactation. **(B)** Content of gut SCFAs in mice during lactation. **(C)** Clustering heatmap of gut SCFAs in mice during adolescence. **(D)** Content of gut SCFAs in mice during adolescence. The statistic difference of various indicators between two groups was evaluated by two-tailed unpaired student’s T test and When p < 0.05, it is considered statistically significant. The significance levels are represented as: *P<0.05, **P<0.01, ***P<0.001, ****P<0.0001, while ns stands for no significant difference.

During the lactation period, acetic acid is the most abundant SCFAs, followed by propionic acid, and the sum of these two SCFAs accounts for more than 85% of all SCFAs. Compared with control mice, exposure of neonatal mice to sevoflurane resulted in decreased levels of multiple SCFAs in intestinal contents, with statistically significant differences observed in acetic acid, butyric acid, isovaleric acid, and valeric acid(t=2.757,p=0.020)(t=8.646,p<0.0001)(t=4.689,p=0.0009) (t=4.950,p=0.0006) (**Figures 3A, B**). The level of isobutyric acid was zero, so it was not included in the statistical analysis.

During adolescence, acetic acid is the most abundant SCFAs, followed by butyric acid, and the sum of these two SCFAs accounts for more than 70% of all SCFAs. Compared with the control group, the sevoflurane-exposed group showed decreased levels of propionic acid, isobutyric acid, butyric acid, isovaleric acid, and valeric acid, but only the levels of butyric acid and isobutyric acid were statistically significant (t=2.392,p=0.0378)(t=2.417,p=0.036) (**Figures 3C, D**). These data indicate that repeated exposure to sevoflurane during the neonatal period can have a sustained impact on the composition of gut SCFAs, with more severe effects observed in the short term.

### 3.3 Repeated sevoflurane exposure in neonatal mice leads to persistent colon barrier damage

**Figure 4:**
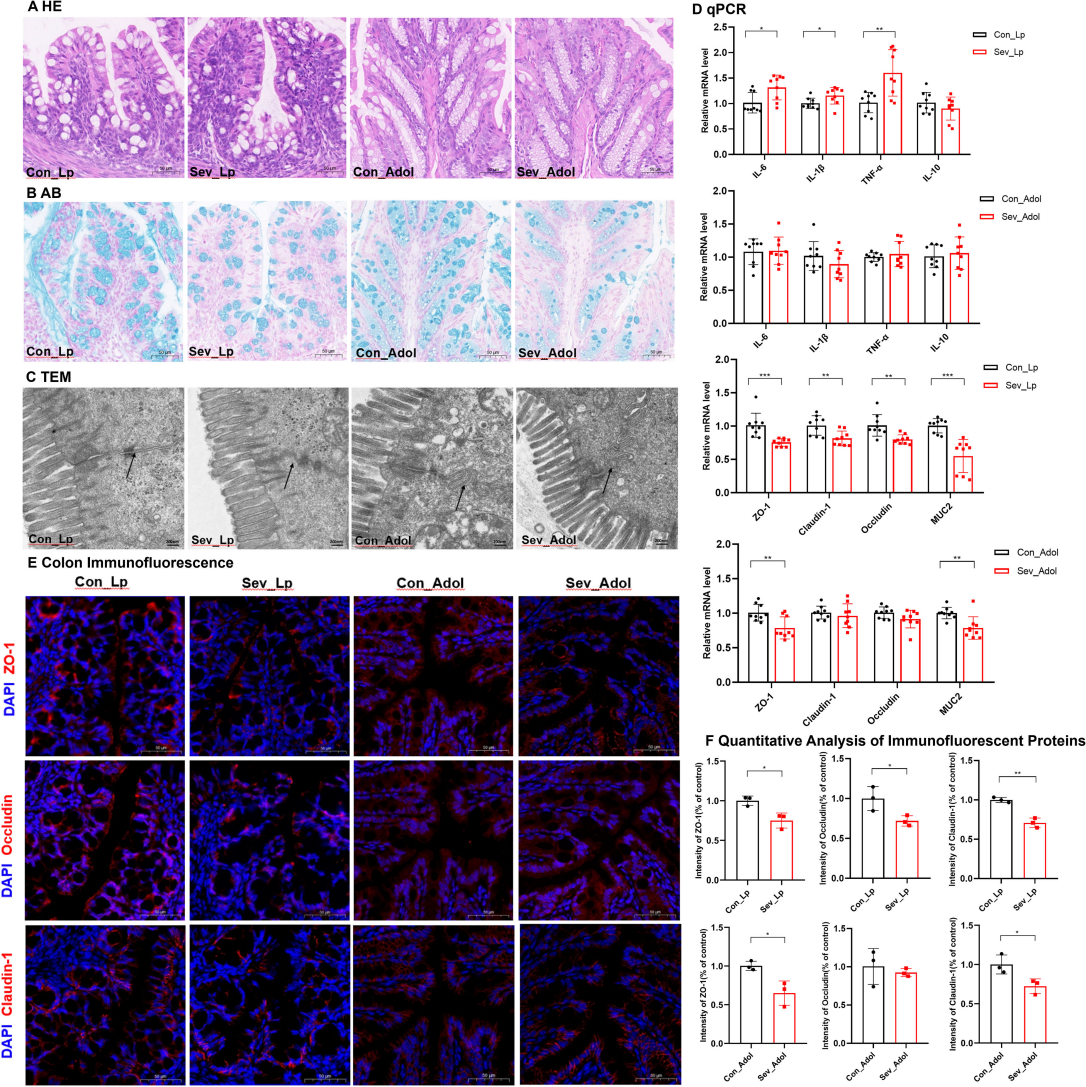
Effects of sevoflurane exposure on colon barrier in neonatal mice during lactation and adolescence **(A)** HE staining showing changes in colon structure; Image scale: 10× magnification. **(B)** AB staining displaying the distribution of mucin and acidic mucopolysaccharides on the colon mucosal surface; Image scale: 10× magnification. **(C)** Transmission electron microscopy revealing the tight junctions in the colon; Image scale: 15,000× magnification. **(D)** qPCR results showing the expression levels of inflammatory factors and colon barrier-related mRNAs in the colon. **(E)** IF staining of colon barrier-related proteins; Image scale: 20x magnification. **(F)** Quantitative analysis of immunofluorescence intensity for colon barrier-related proteins. The statistic difference of various indicators between two groups was evaluated by two-tailed unpaired student’s T test and When p < 0.05, it is considered statistically significant. The significance levels are represented as: *P<0.05, **P<0.01, ***P<0.001, ****P<0.0001, while ns stands for no significant difference.

Through HE staining, we observed that during the lactation period, the sevoflurane-exposed group exhibited significant inflammatory cell infiltration in the mucosal layer, a decrease in the number of goblet cells, submucosal interstitial proliferation, and disordered crypt structure (**Figure 4A**). However, these changes showed some recovery during adolescence. The qPCR results demonstrated that after repeated exposure to sevoflurane, the mRNA expression levels of IL-6, IL-1β, and TNF-α in the colon during lactation were significantly increased (t=2.834, p=0.012) (t=2.344, p=0.032) (t=3.518, p=0.003), respectively. These alterations were mitigated during adolescence (**Figure 4D**), which was consistent with the results of HE staining. There were no significant changes in IL-10 mRNA expression levels either during lactation or adolescence.

AB staining of the colon revealed that in the sevoflurane exposure group, the mucus layer was disrupted and the secretion of new mucus from goblet cells at the basal part was significantly decreased, both during lactation and adolescence (**Figure 4B**). qPCR results indicated that repeated exposure to sevoflurane significantly reduced the mRNA levels of MUC2 in the colonic tissue (t=5.042, p=0.0001), and this effect persisted into adolescence (t=3.563, p=0.0026) (**Figure 4D**).

Further observation of colon microstructure using TEM revealed that sevoflurane exposure during lactation led to blurred tight junctions in colon epithelial cells, while in adolescence, the colon mucosal microvilli became sparse and the tight junctions were discontinuous (**Figure 4C**).

Regarding the colon barrier, sevoflurane exposure significantly decreased the mRNA expression levels of ZO-1, Occludin, and Claudin-1 in the colon during lactation(t=4.066,p=0.0009) (t=3.608,p=0.0024)(t=3.124,p=0.0059). However, during adolescence, only the ZO-1 mRNA expression level was significantly decreased(t=3.284,p=0.0047). There were no significant changes in the mRNA expression level of Reg3r (**Supplementary Figure 1**). Additionally, IF staining showed decreased protein levels of ZO-1, Occludin, and Claudin-1 during lactation (t=3.848, p=0.018) (t=2.967, p=0.041) (t=7.327, p=0.0018), respectively. During adolescence, the protein levels of ZO-1 and Claudin-1 were also decreased (t=3.639, p=0.022) (t=3.148, p=0.034), while there was no statistically significant difference in the protein level of Occludin (**Figures 4E, F**).

These results indicate that repeated sevoflurane exposure in neonatal mice leads to increased colon inflammation, abnormal tissue structure, and impaired intestinal barrier integrity during lactation, and although the degree of intestinal barrier damage recovers somewhat during adolescence, it still persists.

### 3.4 Repeated sevoflurane exposure in neonatal mice leads to impaired myelin development

**Figure 5:**
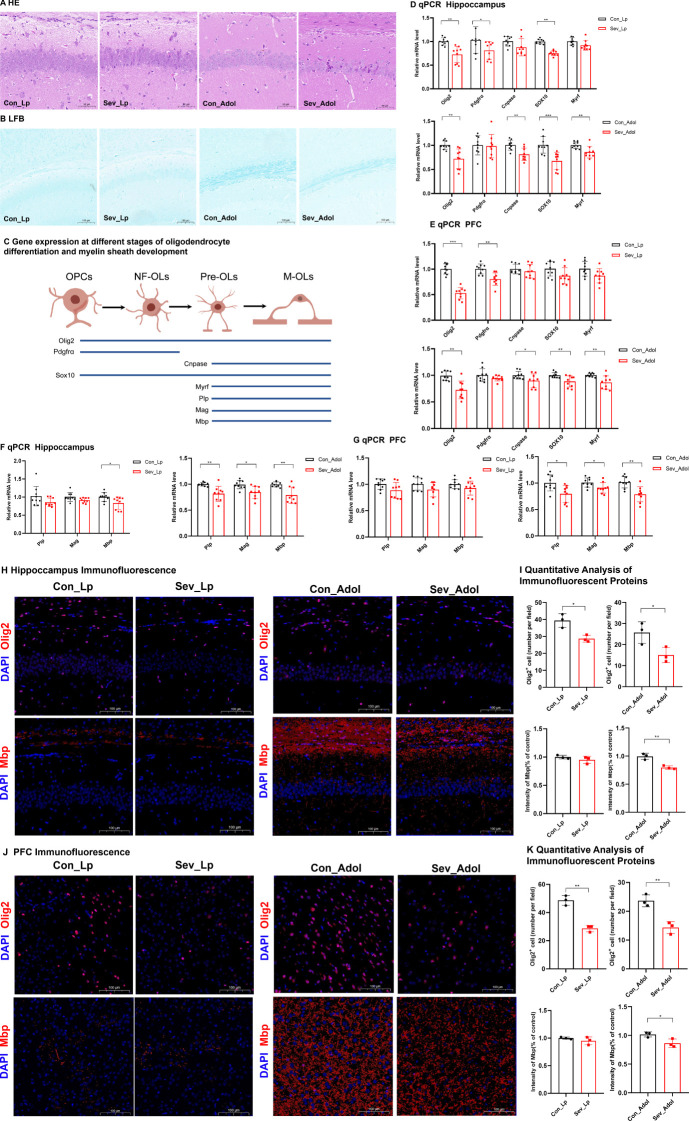
Impaired Oligodendrocyte Differentiation and Myelin Development in Neonatal Mice after Sevoflurane Exposure **(A)** Hematoxylin and Eosin (HE) staining showing changes in neurons in the CA1 region of the hippocampus; Scale bar: 20× magnification. **(B)** Luxol Fast Blue (LFB) staining displaying the myelin structure; Scale bar: 10× magnification. **(C)** Explanation of Gene Expression Related to Different Stages of Oligodendrocyte Differentiation and Myelin Development: Oligodendrocyte Transcription Factor 2 (Olig2), Platelet-Derived Growth Factor Receptor Alpha (Pdgfra), 2’,3’-Cyclic Nucleotide 3’-Phosphodiesterase (Cnpase), SRY-Related HMG-Box 10 (Sox10), Myelin Regulatory Factor (Myrf), Proteolipid Protein (Plp), Myelin-Associated Glycoprotein (Mag), Myelin Basic Protein (Mbp); **(D, E)** Expression levels of genes related to oligodendrocyte differentiation in the hippocampus and prefrontal cortex of mice. **(F, G)** Expression levels of genes associated with myelin development in the hippocampus and prefrontal cortex of mice. **(H, I)** IF detection of Olig2 and counting of Olig2-positive cells in the mouse hippocampus; Scale bar: 20× magnification. IF detection and quantitative results of myelin basic protein in the hippocampus and prefrontal cortex of mice; Scale bar: 20× magnification. **(J, K)** IF detection of Olig2 and counting of Olig2-positive cells in the mouse prefrontal cortex; image scale: 20x magnification. IF detection and quantitative results of Mbp in the mouse prefrontal cortex; image scale: 20x magnification. The statistic difference of various indicators between two groups was evaluated by two-tailed unpaired student’s T test and When p < 0.05, it is considered statistically significant. The significance levels are represented as: *P<0.05, **P<0.01, ***P<0.001, ****P<0.0001, while ns stands for no significant difference.

HE staining revealed that during lactation, the number of neurons in the CA1 region of the hippocampus was significantly reduced in the sevoflurane-exposed group, with some cells showing atrophy, unclear cellular layer structure, condensed and deeply stained cytoplasm, and sparse or missing cells in some areas. This change persisted into adolescence (**Figure 5A**). LFB myelin sheath staining revealed that during lactation, the myelin sheath bands in both groups were relatively obscure. However, during adolescence, the sevoflurane-exposed group exhibited sparse and thinned nerve fibers with lighter staining, indicating the occurrence of myelin sheath damage (**Figure 5B**).

**Figure 5C** demonstrates the expression profiles of relevant genes at different stages of oligodendrocyte differentiation and myelin development. Olig2 is a transcription factor present throughout the entire oligodendrocyte differentiation process. Pdgfrα is one of the markers for oligodendrocyte precursor cells. Cnpase serves as a marker for mature oligodendrocytes. Both Sox10 and Myrf are transcription factors that play crucial roles in glial cell development. Therefore, the expression levels of these genes can serve as indicators of specific developmental stages of oligodendrocytes (Mitew et al., 2014). Plp, Mag, and Mbp are genes associated with myelin development, and the expression of these genes can reflect the status of myelin development.

As shown in **Figures 5D, E**, during lactation, sevoflurane exposure reduced the mRNA expression levels of Olig2, Pdgfrα, and Sox10 in the hippocampus of mice(t=3.393,p=0.005)(t=2.977,p=0.046) (t=3.440,p=0.002), and significantly decreased the mRNA expression levels of Olig2 and Pdgfrα in the prefrontal cortex(t=7.447,p=0.0002)(t=3.794,p=0.0016), with no significant differences in the expression of other genes. These results suggest that repeated sevoflurane exposure in neonatal mice temporarily reduces the number of oligodendrocyte lineage cells and oligodendrocyte progenitor cells. During adolescence, the changes in genes involved in oligodendrocyte differentiation persisted. In both the hippocampus and prefrontal cortex, the mRNA expression levels of Olig2, Cnpase, Sox10, and Myrf were decreased (t=3.808,p=0.0015;t=3.292,p=0.006)(t=3.458,p=0.0032;t=2.289,p=0.0481) (t=4.026,p=0.001;t=2.993,p=0.0086) (t=3.161,p=0.006;t=3.226,p=0.0053), while there was no significant difference in Pdgfrα mRNA expression. As shown in **Figure 5F**, compared to the control group, the experimental group exhibited a significant decrease in Mbp mRNA expression in the hippocampus during both lactation and adolescence(t=2.489,p=0.0242)(t=3.807,p=0.0015), but there were no significant differences in Plp and Mag gene expression during lactation. The expression of Plp and Mag genes in the experimental group during adolescence showed significant decreases (t=3.661, p=0.0021)(t=2.077, p=0.0172). **Figure 5G** shows that during lactation, there were no significant changes in the expression levels of the Plp, Mag, and Mbp genes in the prefrontal cortex, whereas during adolescence, their expression levels were significantly decreased (t=2.80, p=0.0128) (t=2.276, p=0.037) (t=3.850, p=0.0014). This may be due to the fact that myelination in the prefrontal cortex has not yet begun in the early stages.

**Figures 5H–K** presents the IF detection of Olig2 protein in the hippocampus and prefrontal cortex, along with the quantification of protein-positive cells. Exposure to sevoflurane significantly decreased the expression of Olig2 protein in the hippocampal region of mice during lactation and adolescence (t=4.064, p=0.0153) (t=2.946, p=0.0421), as well as in the prefrontal cortex of mice during these same periods (t=8.242, p=0.0012) (t=5.491, p=0.0654), although this latter p-value approached significance. IF analysis with quantitative assessment of mean fluorescence intensity (MFI) for Mbp was conducted. The IF results indicated that during adolescence, the expression of Mbp protein significantly decreased in both the hippocampal region and prefrontal cortex (t=5.487, p=0.0054) (t=3.084, p=0.0368), but there were no significant changes during lactation.

These findings suggest that repeated exposure of neonatal mice to sevoflurane can affect the oligodendrocyte population in the hippocampus and prefrontal cortex in the short term, and this effect continues to develop even after the cessation of the harmful stimulus.

### 3.5 Repeated sevoflurane exposure in neonatal rats induces changes in memory and cognitive behavior

**Figure 6:**
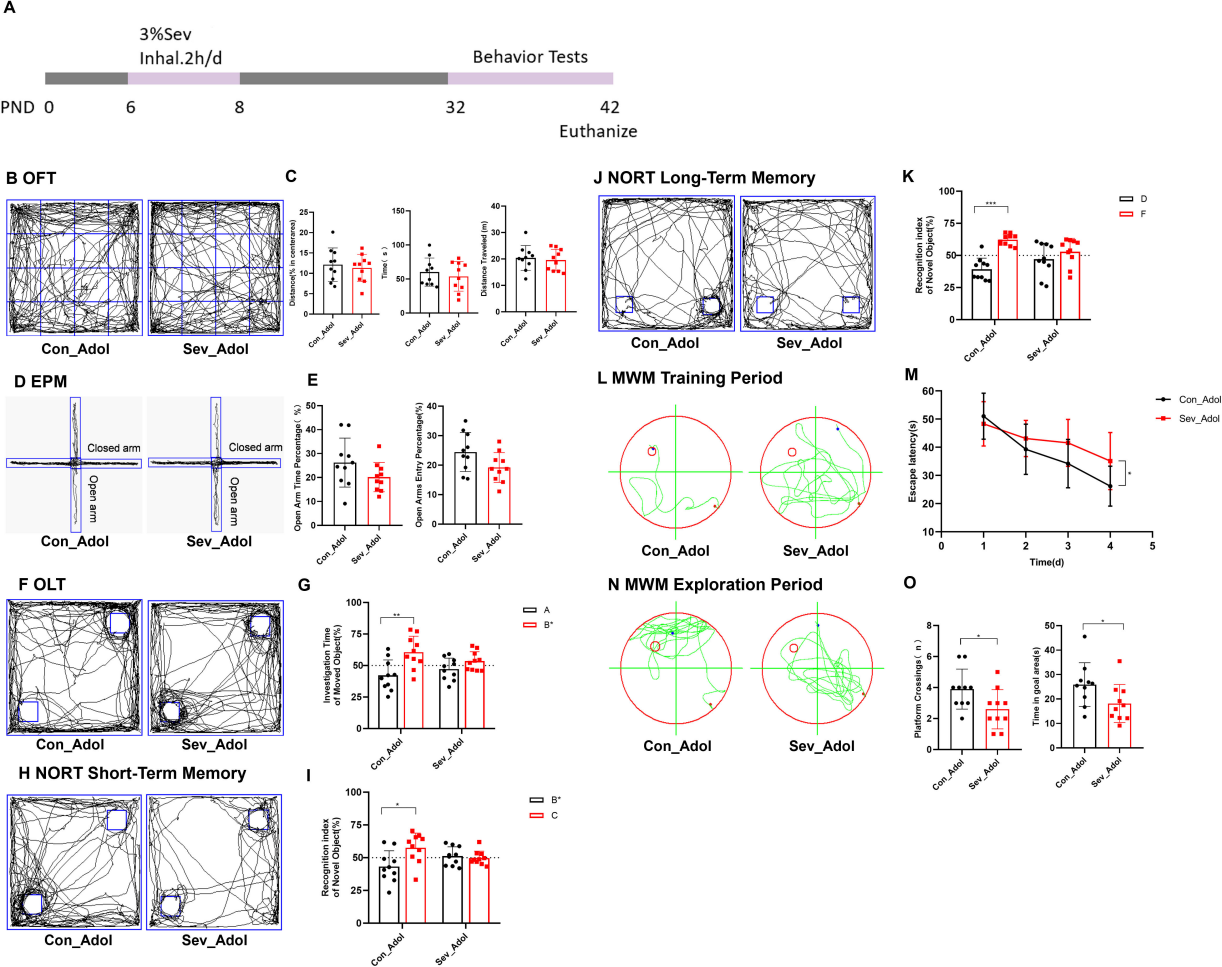
Repeated Sevoflurane Exposure in Neonatal Rats Leads to Memory and Cognitive Behavioral Changes. **(A)** Timeline of sevoflurane exposure and behavioral testing. **(B, C)** Typical movement trajectories of mice in the OFT, along with the percentage of distance traveled, time spent in the central zone, and total distance traveled. **(D, E)** Typical movement trajectories of mice in the EPM, along with the open arm time percentage (OT%) and open arm entry percentage (OE%). **(F, G)** Typical movement trajectories of mice in the OLT, along with the exploration time for the object A in the old location and object B* in the new location. **(H, I)** Typical movement trajectories of mice in the short-term memory experiment of the NORT, along with the exploration time for the old object B* and new object C. **(J, K)** Typical movement trajectories of mice in the long-term memory experiment of the NORT, along with the exploration time for the old object D and new object F. **(L, M)** Typical swimming trajectories of mice during the training phase of the MWM, along with the escape latency. **(N, O)** Typical swimming trajectories of mice during the exploration phase of the MWM, along with the platform crossing frequency and time spent in the target quadrant. The statistic difference of various indicators between two groups was evaluated by two-tailed unpaired student’s T test and When p < 0.05, it is considered statistically significant. The significance levels are represented as: *P<0.05, **P<0.01, ***P<0.001, ****P<0.0001, while ns stands for no significant difference.

As shown in **Figure 6A**, behavioral tests were conducted on mice during the P32-42 period. **Figure 6B** displays the typical movement trajectories of the rats during the OFT. There were no significant differences in the percentage of distance traveled, time spent in the central zone, or total distance traveled between the groups (**Figure 6C**). This indicates that the rats did not exhibit obvious anxiety-like behaviors after sevoflurane exposure. **Figure 6D** displays the typical movement trajectories of the mice in the EPM. Compared with the control group, there were no significant differences in the open arm time percentage (OT%) and open arm entry percentage (OE%) in the sevoflurane-exposed group (**Figure 6E**). Once again, it is noted that no significant anxiety behavior was observed after sevoflurane exposure, which supports the results of the OFT.

**Figure 6F** shows the typical movement trajectories of the rats during the OLT. The control group spent more time exploring the object B* in the new location compared to object A in the old location(t=3.262,p=0.0043), while the sevoflurane-exposed group showed no significant difference in exploration time between the two objects (**Figure 6G**). This suggests that sevoflurane exposure has affected their spatial memory ability. **Figure 6H** displays the typical movement trajectories of the rats in the short-term memory experiment of the NORT. The control group spent more time exploring the new object C(t=2.741,p=0.0134), while the sevoflurane-exposed group showed no significant difference in exploration time between the two objects (**Figure 6I**). **Figure 6J** shows the typical movement trajectories of the rats in the long-term memory experiment of the NORT. The control group spent more time exploring the new object F(t=6.524,p=0.0003), while the sevoflurane-exposed group showed no significant difference in exploration time between the two objects (**Figure 6K**). This indicates that sevoflurane exposure has impacted both their short-term and long-term memory.

**Figure 6L** shows the typical movement trajectories of the mice during the training phase of the MWM experiment. On the fourth day of training, the escape latency of the sevoflurane-exposed group was longer than that of the control group(t=2.276,p=0.0353), indicating that sevoflurane exposure impaired the learning ability of the mice (**Figure 6M**). **Figure 6N** displays the typical movement trajectories of the mice during the exploration phase of the MWM experiment. We found that sevoflurane exposure impaired the spatial working memory and reference memory abilities of the mice, characterized by a significant reduction in platform crossing frequency and time spent in the target quadrant compared to the control group(t=2.278,p=0.0351) (t=2.071,p=0.0530) (**Figure 6O**). Therefore, our research results indicate that repeated sevoflurane exposure in neonatal rats can lead to memory and cognitive impairments in mice.

### 3.6 Relationship between gut microbiota, SCFAs, intestinal physiology, and myelin development as well as behavioral disorders

**Figure 7:**
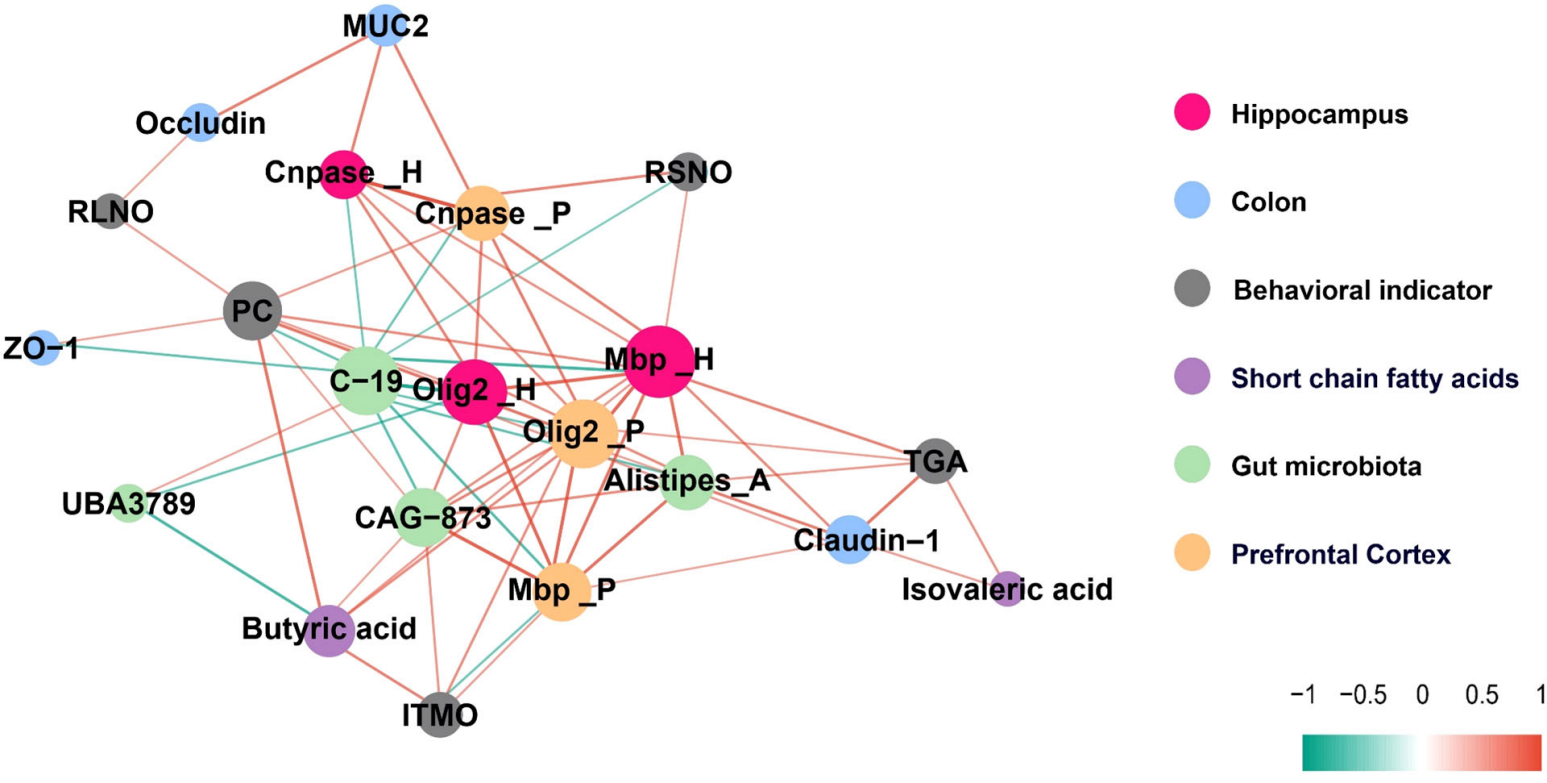
A correlation network diagram illustrating the relationships between gut microbiota, SCFAs, intestinal physiology, myelin development, and behavioral indicators, with |R| ≥ 0.30 and p < 0.05 indicating significance.

To further explore the relationship between gut microbiota, their metabolite SCFAs, intestinal physiology, and the differentiation and maturation of oligodendrocytes in the brain, myelin development, as well as behavioral indicators, this study conducted a relevant network analysis. **Figure 7** illustrates a complex microbe-metabolite-gut-brain interaction in adolescent mice, with |R| ≥ 0.30 and p < 0.05. Our results indicate that myelin development and behavioral changes in mice are closely associated with significantly altered gut microbiota (LDA effect size > 4.5), SCFAs, and colonic physiology. For example, the important transcription factor Olig2 in oligodendrocytes in the hippocampus is negatively correlated with the gut bacteria C-19 and UBA3789. Mbp in the hippocampus and prefrontal cortex is positively correlated with the gut bacteria CAG-873, Alistipes_A, Butyric acid, and Claudin levels. Cnpase is positively correlated with MUC2. The Investigation Time of Moved Object (ITMO) in the OLT experiment is positively correlated with CAG-873 and Butyric acid. The decrease in Platform Crossings (PC) in the MWM experiment observed in mice repeatedly exposed to sevoflurane is attributed to the reduction of colonic ZO-1 and Butyric acid, as well as an increase in C-19. The increase in Time in Goal Area (TGA) in the MWM experiment is associated with an increase in colonic Claudin-1. Additionally, we also found that the Recognition Index of Short-term Novel Object (RSNO) in the NORT experiment is negatively correlated with C-19. The Recognition Index of Long-term Novel Object (RLNO) is positively correlated with Occludin.

### 3.7 Supplementation with sodium butyrate restores the dysregulation of short-chain fatty acid metabolism induced by sevoflurane exposure

**Figure 8:**
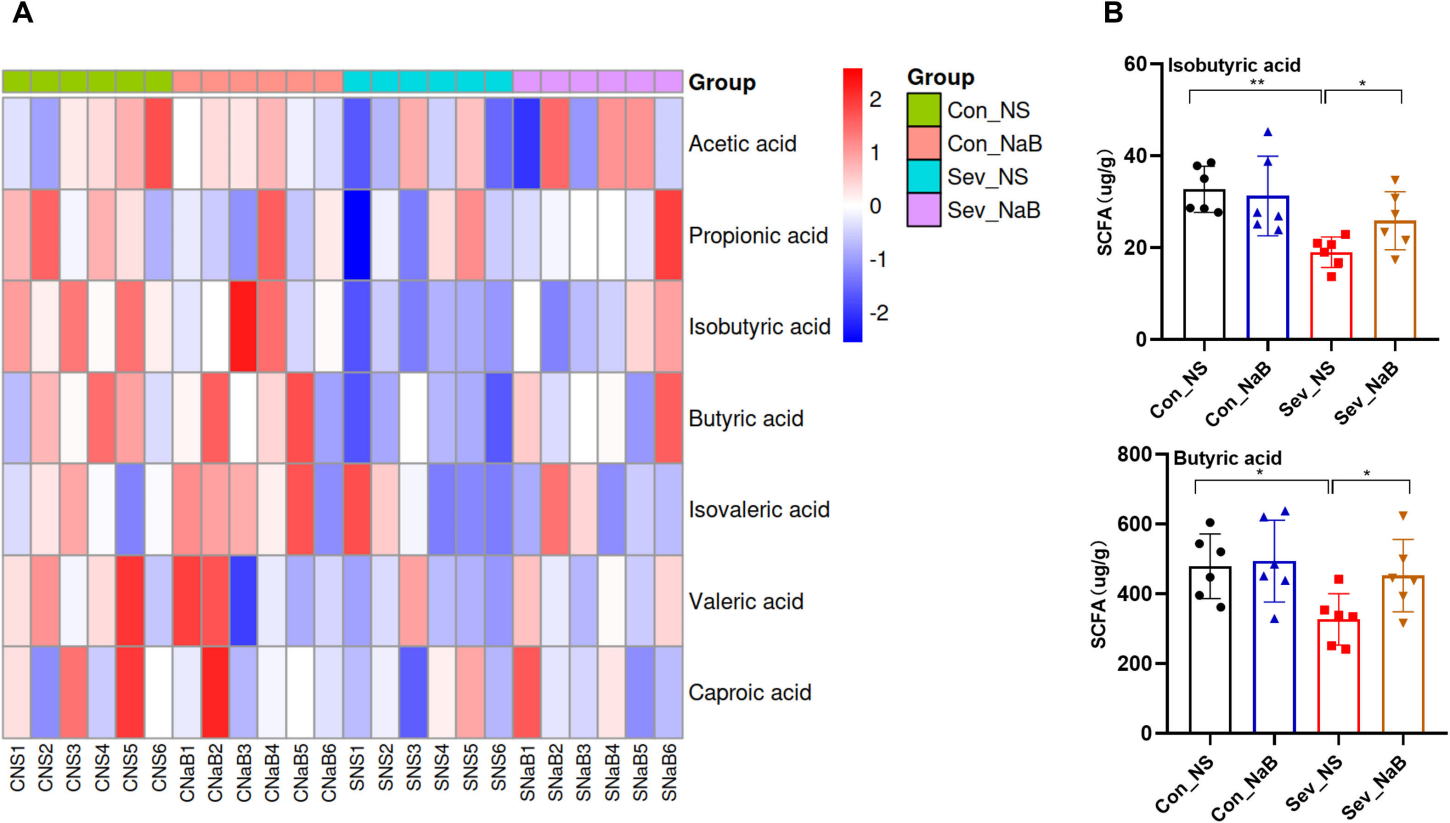
Effects of Sodium Butyrate Treatment on Intestinal SCFAs **(A)** Clustering heatmap of intestinal SCFAs in mice after sodium butyrate treatment. **(B)** Levels of isobutyrate and butyrate in the intestine after sodium butyrate treatment. Statistical differences between groups were evaluated using one-way ANOVA, and multiple comparisons for differences in various indicators between groups were conducted using Tukey’s HSD test. Differences were considered statistically significant when p < 0.05. Significance levels are indicated as follows: *P < 0.05, **P < 0.01, ***P < 0.001, ****P < 0.0001, and ns indicates no significant difference.

There is a significant correlations between butyrate and genes related to oligodendrocyte differentiation, myelin development, as well as certain behavioral alterations (**Figure 7**). To determine whether the intestinal and brain changes observed after sevoflurane exposure could be attributed to decreased butyrate levels, we treated mice with butyrate. **Figures 8A, B** shows that after sodium butyrate treatment, the levels of SCFAs in the intestine were restored, with increased significantly of butyrate and isobutyrate (F=4.139, p=0.039) (F=3.602, p=0.0314).

### 3.8 Supplementation with sodium butyrate ameliorates intestinal barrier damage induced by sevoflurane exposure

**Figure 9:**
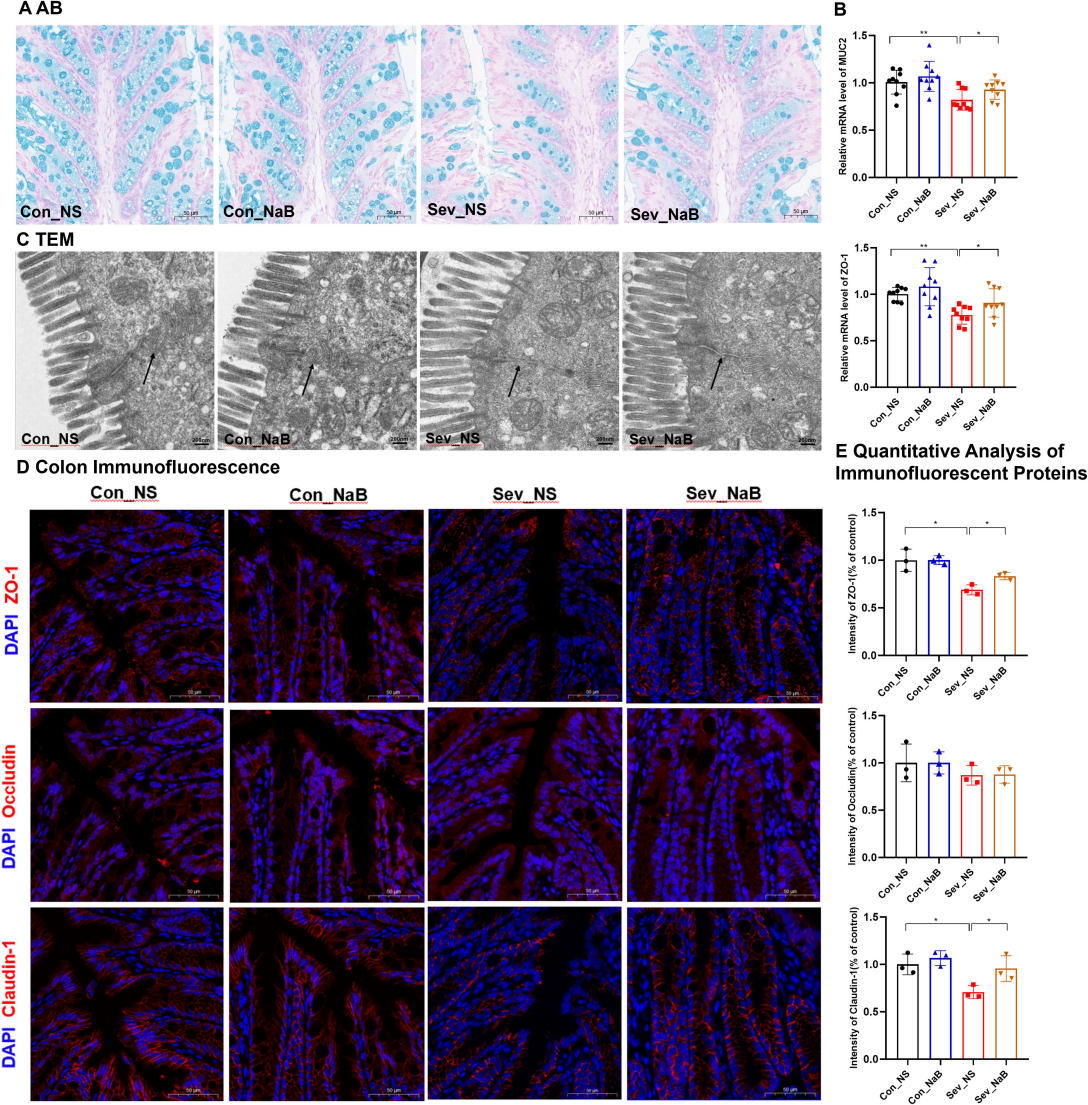
Improvement in Intestinal Physiology of Mice after Sodium Butyrate Treatment **(A)** AB staining showing the distribution of mucin and acidic mucopolysaccharides on the surface of colonic mucosa; scale bar: 10× magnification. **(B)** qPCR results displaying the expression levels of colonic barrier-related mRNAs. **(C)** Transmission electron micrograph showing the tight junctions in the colon; scale bar: 15,000× magnification. **(D, E)** IF detection and quantification results of colon barrier-associated proteins; scale bar: 20× magnification. Statistical differences between groups were evaluated using one-way ANOVA, and multiple comparisons for differences in various indicators between groups were conducted using Tukey’s HSD test. Differences were considered statistically significant when p < 0.05. Significance levels are indicated as follows: *P < 0.05, **P < 0.01, ***P < 0.001, ****P < 0.0001, and ns indicates no significant difference.

Through AB staining, we observed that the amount of new mucus secreted from the base of colonic goblet cells in mice was restored after treatment with NaB (**Figure 9A**). Simultaneously, the expression levels of MUC2 mRNA and ZO-1 mRNA in the colon were increased (F=6.563,p=0.0014) (F=6.764,p=0.0015) (**Figure 9B**).

Further observation of the colonic microstructure by transmission electron microscopy revealed that after NaB treatment, the intestinal mucosal microvilli returned to normal, and the tight junctions were continuous (**Figure 9C**). In terms of the colonic barrier, immunofluorescence detection showed that the expression levels of ZO-1 and Claudin-1 proteins in the colon were partially restored after NaB treatment(F=6.51,p=0.017) (F=7.183,p=0.0117) (**Figures 9D, E**). These results indicate that NaB can, to a certain extent, rescue the impaired colonic barrier integrity in juvenile mice exposed to sevoflurane.

### 3.9 Supplementation with NaB ameliorates myelin development impairment induced by sevoflurane exposure

Following the improvement in short-chain fatty acid levels and intestinal physiology, we re-examined the genes and proteins related to oligodendrocyte differentiation and myelin development in the mouse brain.

**Figure 10:**
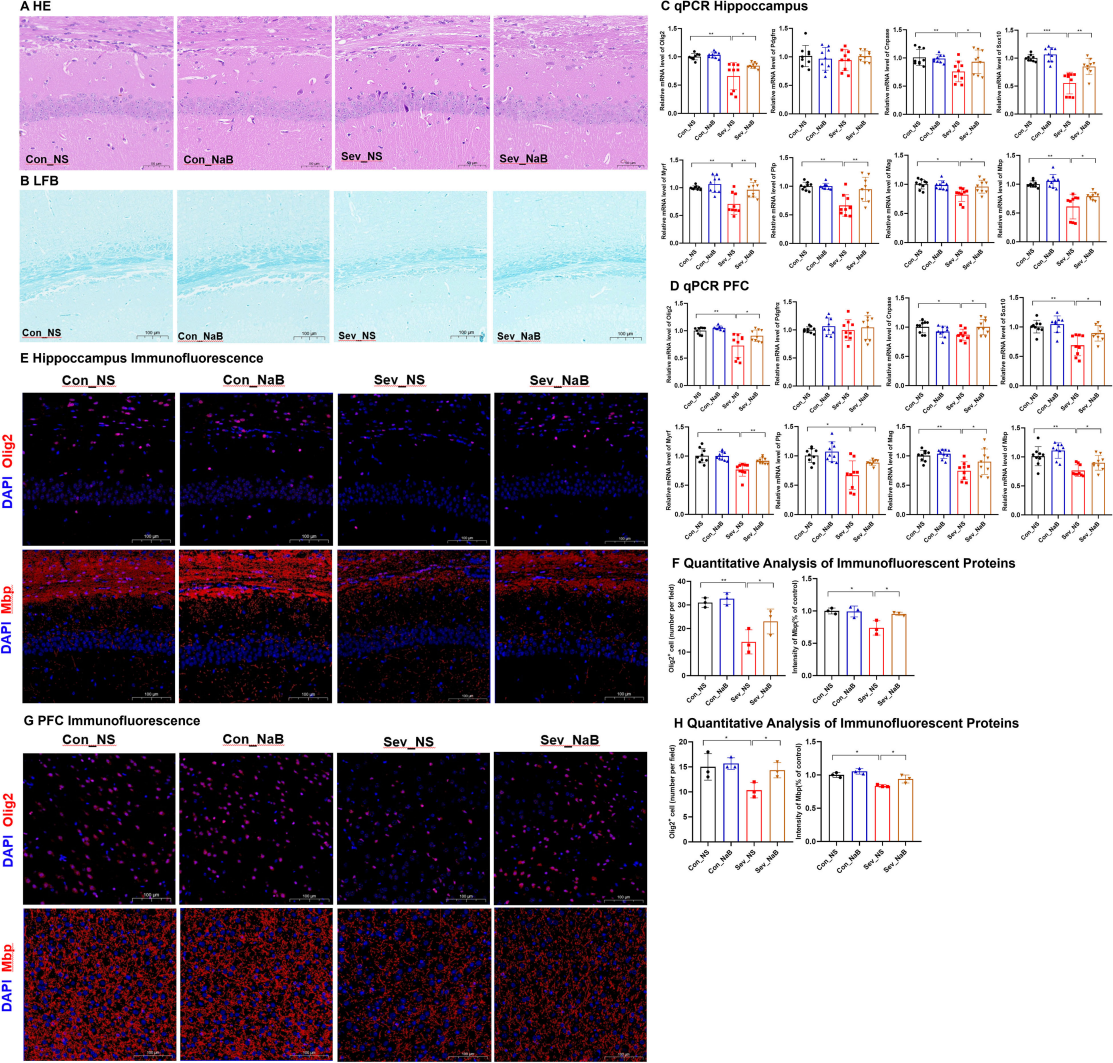
Improvement in Both Intestinal Physiology and Myelin Development in the Brain of Mice after Sodium Butyrate Treatment **(A)** Hematoxylin and Eosin (H&E) staining showing changes in neurons in the CA1 region of the hippocampus; scale bar: 20× magnification. **(B)** Luxol Fast Blue (LFB) myelin staining displaying the myelin structure; scale bar: 10× magnification. **(C, D)** Expression levels of genes related to oligodendrocyte differentiation and myelin development in the hippocampus and prefrontal cortex of mice. **(E, F)** IF detection of Olig2 and protein-positive cell counting in the hippocampus of mice; Image scale: 20x magnification. IF detection and quantification results of MBP in the hippocampus of mice; Image scale: 20x magnification. **(G, H)** IF detection of Olig2 and protein-positive cell counting in the prefrontal cortex of mice; Image scale: 20x magnification. IF detection and quantification results of MBP in the prefrontal cortex of mice; Image scale: 20x magnification. Statistical differences between groups were evaluated using one-way ANOVA, and multiple comparisons for differences in various indicators between groups were conducted using Tukey’s HSD test. Differences were considered statistically significant when p < 0.05. Significance levels are indicated as follows: *P < 0.05, **P < 0.01, ***P < 0.001, ****P < 0.0001, and ns indicates no significant difference.

HE staining reveals that, after NaB treatment, the condition of pyknotic and deeply stained cytoplasm, as well as sparse and missing cells, in the CA1 region of the hippocampus was partially restored (**Figure 10A**). The sparsity and thinning of nerve fibers were also improved (**Figure 10B**). The mRNA levels of Olig2, Cnpase, Sox10, Myrf, as well as Plp, Mag, and Mbp were partially restored in both the hippocampus and prefrontal cortex (F=9.96,p=0.0037;F=7.51,p=0.0029) (F=4.417,p=0.0104;F=3.44,p=0.0282) (F=14.03,p=0.00018;F=8.63,p=0.0026) (F=11.55,p=0.0015;F=9.37,p=0.0048) (F=10.76,p=0.0020;F=4.74,p=0.032) (F=6.358,p=0.0017;F=7.191,p=0.008) (F=12.61,p=0.0011;F=8.35,p=0.0031) (**Figures 10C, D**). Additionally, the expression of Olig2 and Mbp proteins increased in the hippocampus and prefrontal cortex(F=13.13,p=0.0019;F=5.299,p=0.0264) (F=5.354,p=0.0026;F=7.93,p=0.0125) (**Figures 10E–H**).

These results indicate that NaB treatment can, to a certain extent, ameliorate the oligodendrocyte differentiation disorder and myelin development impairment caused by sevoflurane exposure.

### 3.10 Supplementation with sodium butyrate ameliorates memory and cognitive behavioral changes induced by sevoflurane exposure

**Figure 11:**
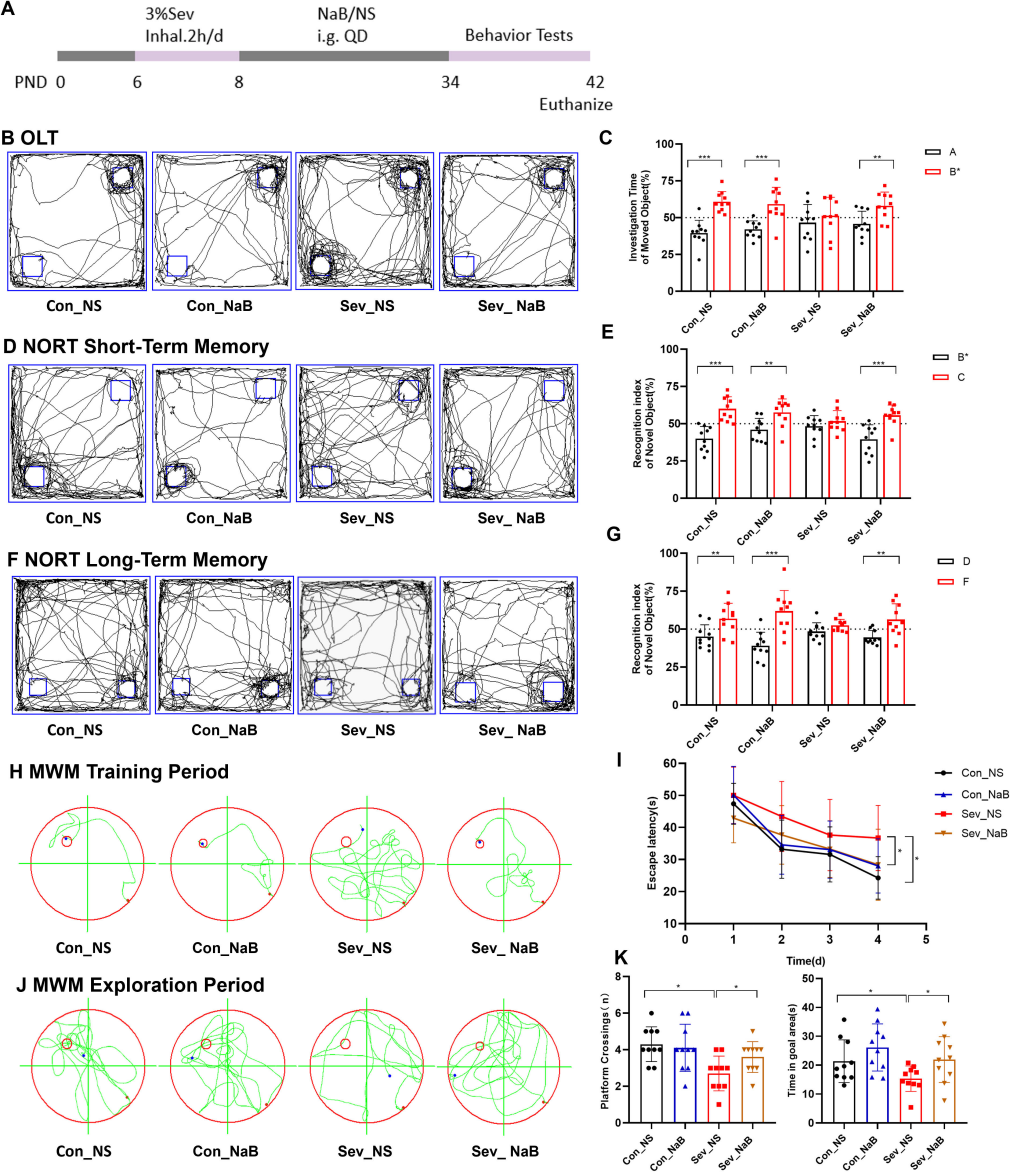
Changes in Memory and Cognitive Behavior of Mice after Sodium Butyrate Treatment **(A)** Timeline of sevoflurane exposure, sodium butyrate treatment, and behavioral testing. **(B, C)** Typical movement trajectories of mice in the OLT and exploration time for the object in the old location A and the object in the new location B. **(D, E)** Typical movement trajectories of mice in the short-term memory test of the NORT and exploration time for the familiar object B and the novel object C. **(F, G)** Typical movement trajectories of mice in the long-term memory test of the NORT and exploration time for the familiar object D and the novel object F. **(H, I)** Typical swimming trajectories of mice during the training phase of the MWM and escape latency. **(J, K)** Typical swimming trajectories of mice during the probe trial of the MWM, along with platform crossing frequency and time spent in the target quadrant. Statistical differences between groups were evaluated using one-way ANOVA, and multiple comparisons for differences in various indicators between groups were conducted using Tukey’s HSD test. Differences were considered statistically significant when p < 0.05. Significance levels are indicated as follows: *P < 0.05, **P < 0.01, ***P < 0.001, ****P < 0.0001, and ns indicates no significant difference.

In the OLT experiment, mice supplemented with NaB showed significant differences in exploration time between the old location object A and the new location object B* (t=3.099,p=0.0062) (**Figures 11B, C**). In the NORT experiment, mice supplemented with NaB exhibited significant differences in exploration time for both the novel objects C and F, as well as the familiar objects B* and D (t=4.259,p=0.0005) (t=3.246,p=0.0045) (**Figures 11D–G**). In the MWM experiment, mice in the NaB-supplemented group demonstrated a significant difference in Escape Latency compared to the non-supplemented group during training, with a notably reduced escape latency in the supplemented group(F=3.224,p=0.0338) (**Figures 11H, I**). Subsequently, on the 5th day when the escape platform was removed, the NaB-supplemented group showed significant differences in platform crossing frequency and time spent in the target quadrant compared to the non-supplemented group (F=4.888,p=0.0119) (F=3.892,p=0.0166) (**Figures 11J, K**). Therefore, our results indicate that supplementation with sodium butyrate partially restores memory and cognitive impairments induced by sevoflurane exposure in young mice.

## 4 Discussion

The formation of the MGBA begins in early life, with the myelination process closely paralleling the establishment of the MGBA (de Faria et al., 2021), Current research has shown that exposure to harmful environmental factors during this period disrupts the balance of this developmental process, inducing neurodevelopmental disorders (Jessen and Mirsky, 2008; Li et al., 2023). Zhou et al. found that exposure to sevoflurane during early growth leads to gut microbiota dysbiosis in rodents (Zhou et al., 2023). Wu et al. discovered that exposure of neonatal rats to sevoflurane disrupts oligodendrocyte maturation and myelination (Wu et al., 2020). These studies suggest that early-life exposure to sevoflurane affects both gut microbiota and myelin development. However, the relationship between these two has not been fully explored. Here, we reveal for the first time that early postnatal repeated exposure to the inhalational anesthetic sevoflurane leads to long-term disruptions in the gut microbiota, reducing the content of SCFAs in the intestine and impairing intestinal barrier function. This exposure also inhibits oligodendrocyte differentiation and myelin development in the hippocampus and prefrontal cortex, subsequently causing memory and cognitive impairments in mice during adolescence. By supplementing with the intestinal metabolite butyrate to restore intestinal SCFAs levels, we can alleviate, to a certain extent, the negative effects of sevoflurane on myelin development and improve long-term memory and cognitive functions.

Serbanescu et al. found that exposure to inhalational anesthetics led to a decrease in gut bacterial diversity, with a reduction in Clostridia and an increase in Proteobacteria and Actinobacteria (Serbanescu et al., 2019). Han et al. reported that exposure to inhalational anesthetics in adult mice resulted in a reduction in the abundance and diversity of the gut microbiota (Han et al., 2021). However, there is a relative lack of literature reporting on the short-term and long-term effects of inhalational anesthetic exposure on the gut microbiota during the early stages of organism growth and development. Our study found that during the suckling period, the gut of mice had low bacterial abundance and diversity, which increased during puberty. Exposure to sevoflurane caused a short-term decrease in gut bacterial diversity in mice, but it recovered during puberty. This indicates that the gut microbiota has a certain capacity for self-recovery and reconstitution, allowing it to undergo a degree of self-adjustment after the removal of external stressors, such as anesthetic exposure. Additionally, in this study, we found that the ratio of Firmicutes to Bacteroidetes (F/B) in the gut of adolescent mice was significantly higher compared to the control group. Studies have shown that an increased F/B ratio in the gut is associated with intestinal inflammation, gut diseases, and some chronic conditions (Yang et al., 2015; Houtman et al., 2022). Another study reported that a deficiency of Omega-3 polyunsaturated fatty acids in the diet of juvenile mice led to an elevated F/B ratio in the gut, accompanied by changes in cognition, anxiety, and social behavior (Robertson et al., 2017), These studies suggest that when the F/B ratio is imbalanced, the gut microecosystem may be disrupted, subsequently affecting the host’s metabolism, immune system, and neural function. In our in-depth exploration of the interplay between gut microbiota and brain function, we utilized LEfSe analysis, Random Forest analysis, and Spearman correlation analysis. We identified significant and characteristic changes in certain bacterial genera that were strongly correlated with memory and cognitive function indicators in the experimental animals. These experimental results indicate that specific changes in the gut microbiota of neonatal mice after multiple exposures to sevoflurane are a notable marker of decreased cognitive ability during puberty. This finding highlights the importance of closely monitoring the dynamic evolution of gut microbiota when assessing and preventing potential neurodevelopmental damage from anesthetic exposure.

SCFAs, which are primary metabolites produced by microbial fermentation activities, play a crucial role in the human body. They provide energy for intestinal epithelial cells, participate in normal metabolic processes, and are involved in regulating gut immunity and barrier function (Hu et al., 2018), They are an indispensable part of maintaining normal bodily functions. Furthermore, studies have shown that SCFAs also have an impact on the central nervous system (Lan et al., 2024). They enter the central nervous system by binding to receptors on the BBB or through other passive pathways, altering gene expression, mitochondrial function, neurotransmitter production, immune activation, and neuronal behavior (Mirzaei et al., 2021). In this study, exposure to sevoflurane caused a significant short-term decrease in SCFAs levels in the gut, particularly in the concentrations of butyrate and isobutyrate, which persisted into adulthood. This may be an important contributor to the disruption of myelin development in the brain. Furthermore, the results of this study indicate that during the lactation period, there were significant inflammatory changes in the gut, accompanied by ongoing damage to intestinal barrier function. Damage to the intestinal barrier not only poses a serious threat to gut health but also serves as a key pathogenic factor in the onset and progression of various diseases both within and outside the gut (Sun et al., 2020; Li et al., 2024). Literature suggests that intestinal barrier damage is associated with certain neurodevelopmental disorders, such as autism and attention-deficit/hyperactivity disorder (ADHD) (Bundgaard-Nielsen et al., 2023), This may be due to the “leaky gut” phenomenon resulting from intestinal barrier damage, which disrupts the signaling between the gut and the brain, leading to abnormal neurodevelopment.

Immature pre-oligodendrocytes, derived from oligodendrocyte precursor cells (OPCs), transition into mature oligodendrocytes, ultimately generating myelin that envelops axons (Crawford et al., 2013). Proper myelination is crucial for axonal conductivity, as well as the development and function of the nervous system. In our study, we found that neonatal mice during the rapid myelination phase, when repeatedly exposed to sevoflurane, exhibited a reduction in the number of oligodendrocyte lineage cells in the hippocampal and prefrontal cortical regions. This was accompanied by disrupted differentiation of oligodendrocyte progenitor cells (OPCs) and decreased myelination, a process that continued even after the cessation of sevoflurane exposure. Specifically, during the lactation period, there was a decline in the number of oligodendrocyte lineage cells (Olig2+ cells) and OPCs (Pdgfrα+ cells). However, in adolescence, while the number of OPCs remained unchanged, there was a notable reduction in the number of oligodendrocyte lineage cells and mature oligodendrocytes. The decrease in OPCs number during lactation without a significant change in adolescence may be attributed to early sevoflurane-induced apoptosis and inhibition of cell proliferation, followed by disrupted OPCs differentiation, leading to an accumulation of precursor cells. Furthermore, our observations also revealed a decrease in the protein levels associated with myelin development, indicating that sevoflurane exposure adversely affects normal myelin development. The prefrontal cortex, a brain region involved in memory and behavioral regulation, is also related to anxiety and depression. The hippocampus participates in learning processes, as well as spatial cognition and memory storage. The loss or damage of myelin in these important brain regions may lead to a series of behavioral abnormalities and cognitive impairments, such as attention deficits and impaired working memory (Preston and Eichenbaum, 2013). Through a series of behavioral experiments, we demonstrated that sevoflurane exposure resulted in long-term memory impairments and learning disabilities in mice. To further explore the relationship between gut microbiota, their metabolite short-chain fatty acids (SCFAs), gut physiology, and brain myelin development, as well as behavioral indicators, this study conducted a Spearman correlation network analysis. The results showed that myelin development and changes in memory and cognitive behavior in mice were closely related to significant alterations in gut microbiota, SCFAs, and colonic physiology.

It is undeniable that sevoflurane, as a general anesthetic, exerts multifaceted and complex effects on brain development. Previous studies have indicated that sevoflurane exposure in neonatal rats can affect OPCs proliferation, migration, differentiation, and myelination by directly causing OPC depolarization and upregulating MT1 receptors (Li et al., 2022; Liang et al., 2021). However, our study demonstrates that intestinal homeostasis is indeed disrupted in juvenile rats exposed to sevoflurane, with a decrease in the gut microbiota product butyrate. Butyrate, a substance produced by beneficial bacteria in the intestine during the process of food digestion, has positive effects on intestinal development, microbial balance, and immune function (Liu et al., 2018). It benefits intestinal physiology, including ion transport and tight junctions (Cresci et al., 2013), and can cross the blood-brain barrier (BBB) to exert positive regulatory effects on the central nervous system (Wei et al., 2023). Butyrate can directly influence oligodendrocytes, promoting the differentiation of immature oligodendrocytes (Chen et al., 2019).

To validate the hypothesis that sevoflurane indirectly affects myelin development by disrupting intestinal homeostasis, we conducted a butyrate supplementation experiment. The experimental results showed that sodium butyrate gavage treatment increased intestinal butyrate levels, optimized the intestinal physiological environment, promoted intestinal mucus secretion, enhanced epithelial cell tight junctions, and restored gene expression related to intestinal barrier function. At the neurodevelopmental level, sodium butyrate also alleviated the sparseness and thinning of nerve fibers, restored gene expression related to oligodendrocyte differentiation and myelin development in the hippocampus and prefrontal cortex, and improved memory and learning abilities in mice. These findings further confirm the crucial role of gut microbiota metabolites in regulating myelin development, and disruptions in this regulatory pathway due to microbiota imbalance can indirectly affect brain development. However, it is noteworthy that although sodium butyrate treatment improved myelin development and behavioral disorders to some extent, it did not achieve complete repair. This suggests that sevoflurane may also damage myelin development through other pathways, such as inducing neuroinflammation. Therefore, subsequent research will delve deeper into the multifaceted mechanisms of sevoflurane’s effects on myelin development, providing more ideas and strategies for the prevention and treatment of clinically relevant diseases.

Furthermore, it is an unavoidable fact that this study indeed has some limitations and shortcomings. Our research is constrained by the use of a rodent model. Although mouse models share many similarities with humans, the differences between the two cannot be ignored, especially in terms of neuronal development and drug metabolism. In the current study, the exposure of mice to sevoflurane during the P6-8 stage has become a commonly used model for exploring the effects of inhalational anesthetics on neurodevelopment (Zhang et al., 2019). However, it should be noted that there may be differences in the developmental stages and characteristics of the neonatal brain compared to those of mice, so the mouse model may not fully simulate the real-world situation after neonatal anesthetic exposure. Moreover, neonatal anesthesia often involves the combined use of multiple drugs as well as different surgical and medical procedures, and these complex factors are difficult to fully replicate in mouse models. In light of this, we look forward to more refined animal models in the future to facilitate deeper scientific exploration.

## 5 Conclusion

**Figure 12:**
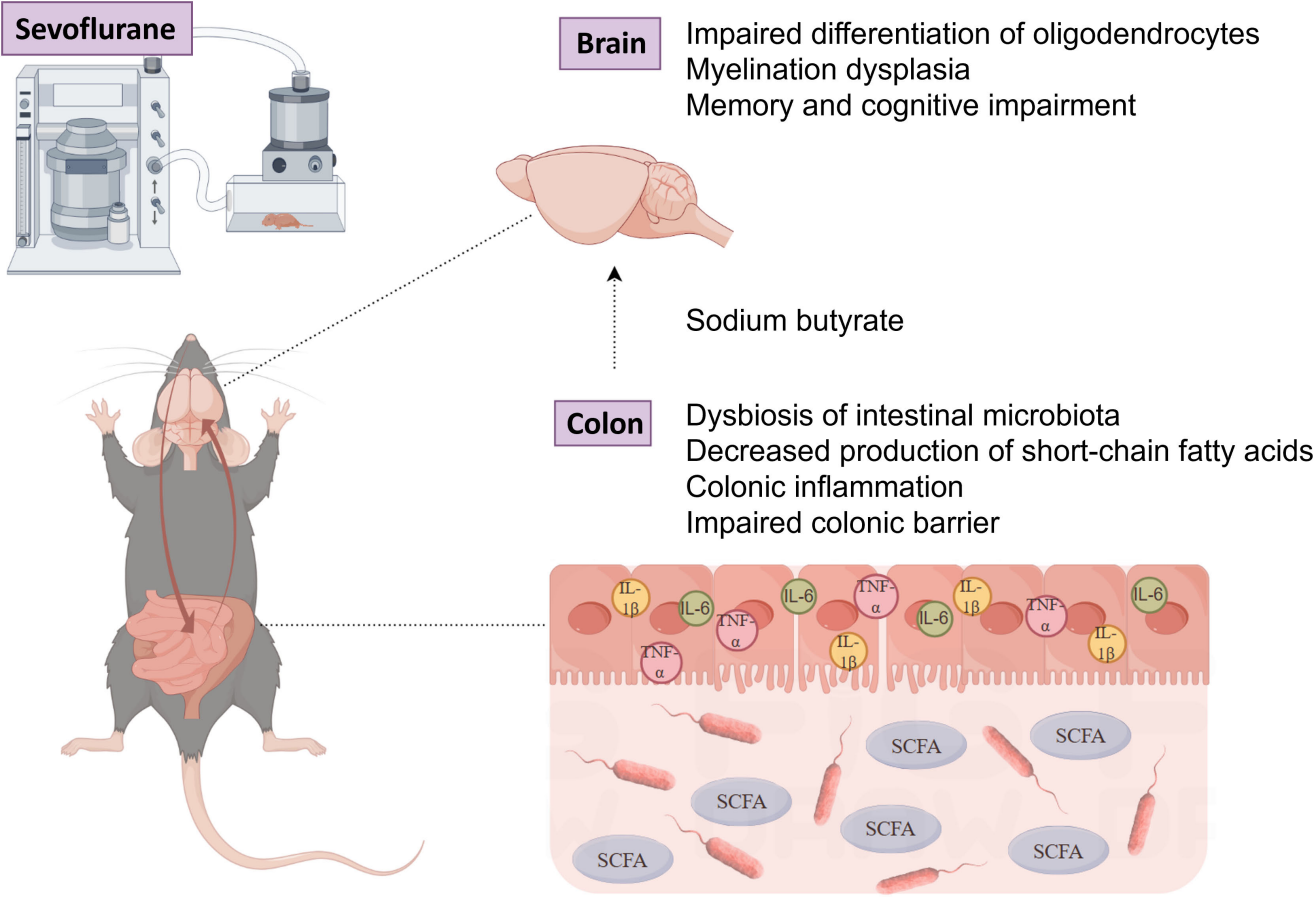
The potential gut-brain mechanism: Exposure of neonatal mice to sevoflurane disrupts the gut microbiota and reduces short-chain fatty acids, which negatively impacts myelin development and behavioral function in the mouse brain. (Illustrated by Figdraw).

Overall, the research results indicate that exposure of neonatal rats to sevoflurane leads to long-term gut microbiota disruption, decreased SCFAs, and intestinal physiological damage. This disordered state of the gut further affects the brain, impeding myelin development and ultimately inducing memory and behavioral disorders. By supplementing with sodium butyrate, these detrimental effects are partially ameliorated (**Figure 12**). This finding emphasizes the pivotal role of gut microbiota and their metabolites during the neonatal period in neurodevelopment. Therefore, in clinical practice, we recommend closely monitoring intestinal changes in neonates, which may provide new perspectives and strategies for preventing and treating the potential neurodevelopmental effects of inhalational anesthetics.

## Data Availability

The data presented in the study are deposited in the NCBI SRA repository, accession number PRJNA1230994.
