# Supplementary material for: The role of intestinal homeostasis in sevoflurane-induced myelin development and cognitive impairment in neonatal mice
Source: Front Cell Infect Microbiol. 2025 Mar 12;15:1541757. doi: 10.3389/fcimb.2025.1541757 (PMC11936920; doi:10.3389/fcimb.2025.1541757)

| **Table 1 The antibodies used in this study** | | | |
| --- | --- | --- | --- |
| Antibody | Dilution | 货号 | Source |
| Olig2 | 1：1000 | AB109186 | Abcam，UK |
| MBP | 1：100 | AB209328 | Abcam，UK |
| ZO-1 | 1：500 | AB307799 | Abcam，UK |
| Occludin | 1：100 | AB216327 | Abcam，UK |
| Claudin-1 | 1：1000 | AB211737 | Abcam，UK |
| Goat anti-rabbit IgG | 1：500 | GB25303 | Servicebio，China |
| Goat anti-mouse IgG | 1：500 | GB25301 | Servicebio，China |

| **Table 2 The specific primer pairs used in polymerase chain reaction.** | | |
| --- | --- | --- |
| Gene | Forward (5′-3′) | Reverse (5′-3′) |
| Myrf | TCAGAGGCCATCGTTCAG | CCCAGTCAAGGGAAGCA |
| Mag | CCTTTCCAGGGAGCACA | AGCACACAATGGCAATCAG |
| Plp | GGCACTTCCAACTGAGGA | CCAGGGACATTTCTGCTCT |
| Olig2 | GTGGCCTTCCCTCCTGT | GCTTGCTCCTGTGCTCTG |
| Sox10 | ACGGTTTTCCACTTCCTCA | GTCTTGTTCCTCGGCCAT |
| Pdgfrα | GCTCGAAGTCAGATCCACA | GCATCCTCAGCCCCTAC |
| Cnpase | CATCAGGGTTCTTGTGCTT | GCTTCTTCAGGTCATCGG |
| Mbp | TCCCAAGGCACAGAGACA | CTAAAGAAGCGCCCGATG |
| Claudin-1 | TGAGTTCCCTCCCTTTGC | CCCGTACCTTCCTTCCG |
| Reg3γ | CTTCCTTCCTGTCCTCCA | CCACTCCCATCCACCTC |
| Muc2 | ACATGGATGGCTGCTTCT | TGCTCACAGTCGTTGGTAA |
| Il10 | AGGGTTACTTGGGTTGCC | GGGTCTTCAGCTTCTCACC |
| Il 6 | AGCCCACCAAGAACGATAG | GGTTGTCACCAGCATCAGT |
| Il 1β | AGTTGACGGACCCCAAA | TCTTGTTGATGTGCTGCTG |
| Tnf-α | CGCTGAGGTCAATCTGC | GGCTGGGTAGAGAATGGA |
| Occludin | CTGCCTGCACGATGT | GAGTGTTCAGCCCAGTCAA |
| ZO-1 | ACCATGCCTAAAGCTGTCC | GGAACTCAACACACCACCA |
| Actb | TCTTTGCAGCTCCTTCGT | GACCCATTCCCACCATC |

**Figure.1**


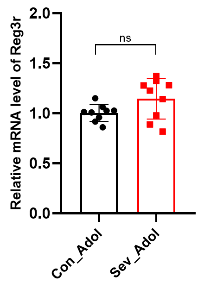

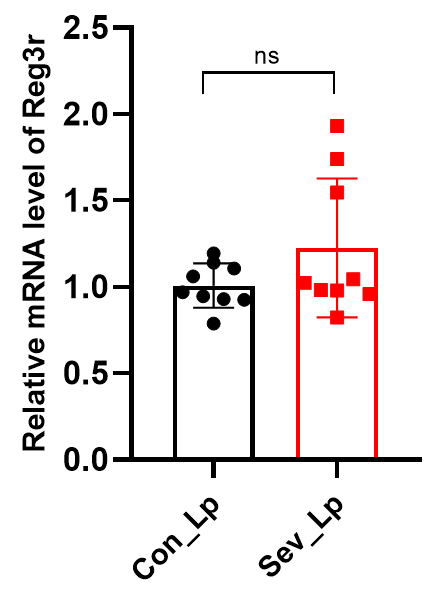

Supplement: Supplementary file 1 [file DataSheet1.docx]
